# Elevated Kallikrein-binding protein in diabetes impairs wound healing through inducing macrophage M1 polarization

**DOI:** 10.1186/s12964-019-0376-9

**Published:** 2019-06-10

**Authors:** Juan Feng, Chang Dong, Yanlan Long, Lifang Mai, Meng Ren, Lingyi Li, Ti Zhou, Zhonghan Yang, Jianxing Ma, Li Yan, Xia Yang, Guoquan Gao, Weiwei Qi

**Affiliations:** 1 Program of Molecular Medicine, Affiliated Guangzhou Women and Children’s Hospital, Zhongshan School of Medicine, Sun Yat-sen University, Guangzhou, China; 2 Department of Biochemistry, Zhongshan School of Medicine, Sun Yat-sen University, 74 Zhongshan 2nd Road, Guangzhou, 510080 China; 3 Department of Endocrinology, the Second Affiliated Hospital, Sun Yat-sen University, Guangzhou, 510030 China; 4 Department of Physiology, University of Oklahoma Health Sciences Center, Oklahoma City, OK 73104 USA; 5 Guangdong Engineering & Technology Research Center for Gene Manipulation and Biomacromolecular Products, Sun Yat-sen University, Guangzhou, China; 6 Guangdong Province Key Laboratory of Brain Function and Disease, Zhongshan School of Medicine, Sun Yat-sen University, Guangzhou, China; 7 School of stomatology and medicine, Foshan University, Foshan, 528000 China

**Keywords:** Diabetic wound healing, Kallikrein-binding protein, Monocyte-macrophages, Notch/NF-κB signalling

## Abstract

**Background:**

The accumulation of M1-polarized macrophages and excessive inflammation are important in the pathogenesis of diabetic foot ulcer (DFU). However, the underlying mechanism of DFU pathogenesis and the crucial regulators of DFU are less well known. Our previous study reported that kallikrein-binding protein (KBP), an angiogenesis inhibitor, was significantly upregulated in diabetic patients compared to its levels in controls. The effects of KBP on monocyte chemotaxis and macrophage M1 polarization were elucidated in this study.

**Methods:**

Plasma KBP levels and monocyte counts were assessed by ELISA and flow cytometry. Wound closure rates in different groups were monitored daily. The phenotype and recruitment of macrophages were measured by real-time PCR, western blot and immunofluorescence assays. The expression of Notch and NF-κB signalling pathway members was determined by the methods mentioned above. ChIP and dual-luciferase reporter gene assays were employed to explore the binding and transcriptional regulation of Hes1 and iNOS.

**Results:**

We found that plasma KBP levels and circulating monocytes were elevated in diabetic patients compared to those in nondiabetic controls, and both were higher in diabetic patients with DFU than in diabetic patients without DFU. KBP delayed wound healing in normal mice; correspondingly, KBP-neutralizing antibody ameliorated delayed wound healing in diabetic mice. Circulating monocytes and macrophage infiltration in the wound were upregulated in KBP-TG mice compared to those in control mice. KBP promoted the recruitment and M1 polarization of macrophages. Mechanistically, KBP upregulated iNOS by activating the Notch1/RBP-Jκ/Hes1 signalling pathway. Hes1 downregulated CYLD, a negative regulator of NF-κB signalling, and then activated the IKK/IκBα/NF-κB signalling pathway.

**Conclusions:**

Our findings demonstrate that KBP is the key regulator of excessive inflammation in DFUs and provide a novel target for DFU therapy.

**Electronic supplementary material:**

The online version of this article (10.1186/s12964-019-0376-9) contains supplementary material, which is available to authorized users.

## Background

Diabetic foot ulcer (DFU) is one of the most intractable complications of diabetes mellitus and leads to nontraumatic amputation in more than 70,000 patients worldwide [1, 2]. The pathological impairment of wound healing is the foremost reason for DFU. Wound healing consists of the following overlapping dynamic phases: inflammation, re-epithelization and neovascularization, and tissue remodelling [3, 4]. The local inflammatory response initiated during wound healing includes the migration and proliferation of diverse cells in addition to the regulation of inflammatory factors and cytokines [3].

Macrophages, which derive from monocytes and upstream progenitor cells, are involved in all the phases of wound healing [5]. Macrophage colony-stimulating factor (M-CSF) and monocyte chemoattractant protein-1 (MCP-1) are vital cytokines for macrophage survival, differentiation and mobilization [5–7]. Furthermore, the recruitment of monocyte-macrophages to wounds depends on MCP-1 secreted by diverse skin cells and the expression of its receptor chemokine receptor 2 (CCR2) on monocyte-macrophage surfaces [5, 8]. Macrophages 2assume a spectrum of activation states ranging from pro-inflammatory M1 macrophages that induce an inflammatory response with the secretion of inflammatory factor [9] to anti-inflammatory M2 macrophages that promote the absorption of inflammation and wound healing [10–12]. M1 macrophages are characterized by the production of inflammatory mediators, such as inducible nitric oxide synthase (iNOS), IL-6, IL-12, and TNF-α, in response to IFN-γ and LPS [13]. M2 macrophages express anti-inflammatory mediators; promote angiogenesis mediators, such as arginase-1 (ARG1), IL-10, TGF-β1, and VEGF; and play a pivotal role in tissue repair, reconstruction and tumours [14, 15]. Delayed diabetic healing is characterized by excessive inflammation with the prolonged accumulation of M1 macrophages and elevated pro-inflammatory cytokines. In addition, anti-inflammatory factors and growth factors secreted by M2-polarized macrophages are also downregulated [16]. However, the reason for this abnormal phenotypic transformation in M1/M2 macrophages in diabetic patients is not well defined.

Kallikrein-binding protein (KBP), also named *SERPINA3K*, was originally identified as a member of the serine proteinase inhibitor (serpin) family [17]. KBP is a plasma protein mainly synthesized and secreted by the liver that has a wide-ranging spectrum of activities, including the relaxation of blood vessels and the inhibition of angiogenesis and antioxidative stress [18, 19]. Our previous studies have shown that circulating KBP levels are increased in diabetic patients with microvascular complications compared to those in diabetic patients without microvascular complications; furthermore, KBP delays diabetic wound healing through inhibiting angiogenesis [20, 21]. Although the effect of KBP on angiogenesis in diabetic wound healing has been reported [21], the effects of KBP on macrophage polarization and the excessive inflammatory reaction in diabetic wound healing have not been documented.

The Notch family is a family of evolutionarily conserved proteins that regulate cell differentiation, proliferation, survival and development [22]. Notch ligands bind with their receptors, resulting in intramembranous cleavage by γ-secretase to release Notch intracellular domain (NICD). NICD translocates into the nucleus and binds to the DNA-binding protein RBP-Jκ to activate Notch target genes, such as Hes1 and Deltex [22]. Notch signalling plays a pivotal role in regulating the development and differentiation of monocyte-macrophages [23, 24]. Nevertheless, the role of KBP in regulating monocyte-macrophages through Notch signalling during wound healing has not been verified.

The NF-κB signalling pathway is a classic pathway that promotes the M1 polarization of macrophages [13]. The activation of inhibitor of κBα (IκBα) kinase (IKK) promotes the phosphorylation of IκBα, which is the inhibitory form of IκBα, following which NF-κB p65 is activated and translocated into the nucleus to activate the expression of target genes [25, 26]. The activation of the Notch signalling pathway can promote the activation of the NF-κB signalling pathway [27–30]. It remains to be explored whether KBP promotes the M1 polarization of macrophages via activating the Notch and NF-κB signalling pathways.

In this study, we elucidated the role of KBP in the excessive inflammatory response during diabetic wound healing. We additionally tested the hypothesis that KBP regulates the numbers and polarization of monocyte-macrophages by activating the Notch and NF-κB signalling pathways, consequently delaying wound healing.

## Materials

### Human subjects

The collection of human samples adhered to the Declaration of Helsinki and was approved by the Ethics Committee of Sun Yat-sen Memorial Hospital. All patients provided their informed consent. All diabetic patients with or without DFU were diagnosed by a medical doctor.

### Animal experiments

All the animal experiments were carried out with the approval of the Animal Care and Use Committee of Sun Yat-sen University (approval ID: SCXK 2011–0029). Wild-type C57BL/6 mice were purchased from the Laboratory Animal Center of Sun Yat-sen University. A human KBP transgenic C57BL/6 mouse strain (KBP-TG) generated as previously described was provided as a gift from Dr. Jianxing Ma (University of Oklahoma Health Sciences Center) [21]. Six-week-old male mice were fed a high-fat diet (60% of calories, D12492, Research Diets, Inc.) for one month and then intraperitoneally injected with streptozotocin (STZ; 40 mg/kg/day) daily for 7 days to induce type 2 diabetes [4, 31, 32]. The type 2 diabetic mice were randomly divided into two groups: an IgG group and a KBP antibody (0.4 mg/kg/day) group. IgG (Sigma-Aldrich, St. Louis, MO, USA) or KBP-neutralizing antibody (Genscript, China) was intraperitoneally administered to the diabetic mice every day beginning three days before the establishment of a wound model for 15 days. BSA or KBP (20 mg/kg/day) was intraperitoneally administered to the WT mice every day beginning three days before the establishment of a wound model for 15 days. Male db/db mice, which is also a type 2 diabetes mouse model, were purchased from Nanjing Model Animal Center. Wound healing rates were observed, the wounds were photographed every other day, and wound tissues from different mouse models were collected.

### Cell culture

Bone marrow-derived macrophages (BMDM) were generated as previously described [33]. BMDMs and mouse RAW264.7 macrophages were cultured in DMEM with 10% FBS and 1% penicillin/streptomycin. THP-1 cells were cultured in RPMI-1640 with 10% FBS and 1% penicillin/streptomycin. THP-1 cells were differentiated with phorbol 12-myristate 13-acetate (PMA) (20 ng/mL, Sigma) for 72 h.

### ELISA to detect KBP, GM-CSF/M-CSF, TNFα, IL-6 and MCP-1

The plasma level of KBP was detected using a human KBP ELISA kit (R&D Systems, Minneapolis, MN, USA, #DY1669) according to the manufacturer’s instructions. The levels of GM-CSF, M-CSF, MCP-1, TNFα and IL-6 in mouse plasma or cellular supernatants were measured with a mouse GM-CSF ELISA kit (R&D Systems, #MGM00), mouse M-CSF ELISA kit (RayBiotech, RayBiotech, Norcross, GA, USA, #ELM-MCSF-1), mouse MCP-1 ELISA kit (RayBiotech, #ELM-MCP-1), mouse TNFα ELISA kit (R&D Systems, #DY410–05) and mouse IL-6 ELISA kit (R&D Systems, #DY406–05).

### Wound healing assays

The dorsa of anaesthetized mice were clipped to remove hair, and then standardized circular wounds were made with a full-thickness 6-mm biopsy punch (Acuderm, Fort Lauderdale, FL). Wound closure rates were monitored by tracing the wound area daily through photographs that were quantified with ImageJ software. Frozen wound tissue slides were stained with F4/80 antibody (1:200, Abcam, Cambridge, MA, USA, #ab6640), iNOS antibody (1:200, Abcam, #ab3523) or ARG1 antibody (1200, Santa Cruz, CA, USA, sc-20,150).

### RNA extraction, reverse transcription of cDNA, and real-time quantitative PCR

RNA extraction, reverse transcription and real-time PCR were performed as described previously [34]. The primers used for real-time PCR are listed in Additional file 1: Table S2.

### Western blotting

Western blot analysis was performed as described previously [4, 35]. The proteins were transferred to a PVDF membrane (Millipore, Billerica, MA, USA) and probed with primary antibodies specific for iNOS (1:1000, Abcam, #ab3523), ARG1 (1:200, Santa Cruz, sc-20,150), Notch1 (1:1000, CST, Danvers, MA, USA, #4380S), Hes1 (1:1000, CST, #11988) and β-actin (1:10000, Sigma-Aldrich, #A5441) overnight at 4 °C. The following secondary antibodies were used: goat anti-rabbit IgG/HRP (1:1000, Vector Laboratories, Burlingame, CA, USA, #PI1000) and goat anti-mouse IgG/HRP (1:5000, Vector Laboratories, #PI2000). Chemiluminescence was developed using ECL Western blotting substrate.

### Immunofluorescence staining and immunohistochemistry

For immunofluorescence staining, wound sections were fixed in 4% paraformaldehyde and permeabilized with 0.01% Triton X-100 in PBS. The samples were incubated with F4/80 (1:200, Abcam, #ab6640), iNOS (1:200, Abcam, #ab3523) or ARG-1 (1:200, Santa Cruz, #sc-20,150) antibodies overnight at 4 °C and then incubated with Alexa Fluor 488-donkey anti-rat IgG (H + L) (1:200, Life Technologies, Gaithersburg, MD, USA, #A21208) and Alexa Fluor 594-donkey anti-rabbit IgG (1:200, Life Technologies, #R37119) for 1 h. The slices were digitally photographed with a confocal microscope. For immunohistochemistry, tissue slices were prepared as described before [36]. The sections were incubated with F4/80 antibody overnight at 4 °C and then incubated with a biotin-conjugated secondary antibody for 30 min, followed by incubation with DAB for 10 s and haematoxylin staining for 30 s. The IHC signal for F4/80 was analysed using ImageJ.

### Transwell migration assay

Chemotaxis experiments were performed using 24-well Boyden chambers (Corning, NY) as described previously [4, 37]. Briefly, DMEM containing 10% FBS was placed in the lower chamber. A total of 1 × 10^5^ RAW264.7 cells in 200 μL medium were seeded into the upper chamber. Macrophages were preincubated with 640 nM KBP for 48 h prior to seeding. The chamber was then incubated for 12 h. The number of macrophages that migrated to the lower surface of the membrane was counted in 10 random high-power fields under a light microscope (Nikon Eclipse, USA). Each assay was performed in triplicate wells.

### ChIP (chromatin immunoprecipitation) assay

RAW264.7 cells were grown in a 10-cm dish (90–95% confluence), and histones were crosslinked to DNA with 1% formaldehyde for 15 min at 37 °C. The cells were washed three times with ice-cold PBS and scraped into a tube for nuclear protein extraction using the NE-PERTM reagent kit (Pierce). Subsequent steps were performed as described previously [38]. PCR was performed to amplify fragments of the iNOS promoter using 2 μL of the extracted DNA (with or without antibody) as a template. The primers used to amplify the iNOS promoter were 5′-TGTACATGCAAGGCAAGCAC-3′ and 5′-TGGCCTCAATAGTTGGGAGAAC-3′.

### siRNA transfection

Hes1 siRNA, RBP-Jκ siRNA and control siRNA were purchased from RiboBio. Transfections were performed at approximately 60% confluency using Lipofectamine® 3000 transfection reagent (Invitrogen) according to the manufacturer’s instructions.

### Flow cytometry

To quantify the circulating monocytes by FACS analysis, whole mouse blood cells were collected in anticoagulant tubes, and PE-labelled CD115 antibody was added (1:100, BD); the cells were then incubated at 37 °C in the dark for 1 h. Ten millilitres of red cell lysate was added to the cells for 5 min at room temperature, following which the cells were centrifuged at 2000 rpm for 3 min, the supernatant was removed, and the cells were washed twice with PBS and resuspended in 300 μl PBS for flow cytometry (Beckman Coulter, CytoFLEX). The data were analysed via CytExpert2.0 software and at least 10,000 gated events were acquired from each sample.

### Data analysis

All the data are expressed as the mean ± standard deviation. Student’s *t*-test was applied for comparisons between two groups, and one-way ANOVA followed by LSD *t*-test was used to compare differences between more than two different groups (GraphPad Prism software). A *P* value less than 0.05 indicated statistical significance.

## Results

### Elevated KBP and monocyte counts in diabetic patients with diabetic foot ulcer

**Fig. 1:**
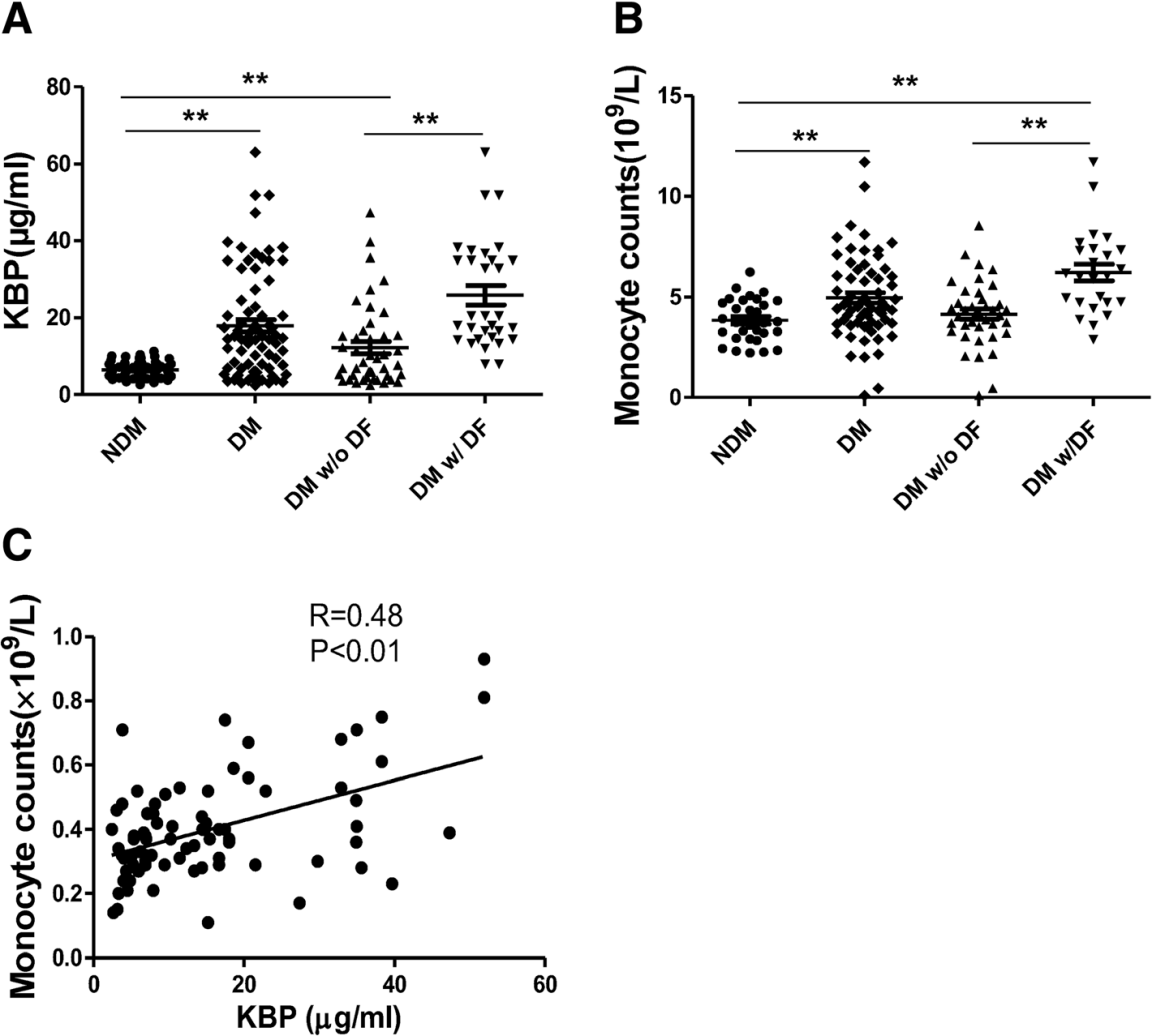
Clinical data and the role of KBP in wound healing. **a** The plasma level of KBP in NDM, DM, DM w/o DFU and DM w/ DFU patients. **b** Monocyte counts in the blood of NDM, DM, DM w/o DFU and DM w/ DFU patients. The collected data refer to the clinical data detected by a blood cell analyser. **c** The correlation of KBP and monocyte counts in the patients in all groups. NDM, *n* = 61; DM, *n* = 69; DM w/o DFU, *n* = 44; DM w/ DFU, *n* = 25

The level of KBP in plasma samples from 61 nondiabetic controls (NDM), 44 diabetic patients without diabetic foot ulcer (DM w/o DFU) and 25 diabetic patients with diabetic foot ulcer (DM w/DFU) was examined by ELISA. The clinical data, such as age, monocyte counts and some biochemical indicators, were analysed (Additional file 1: Table S1). We found that the plasma level of KBP in the DM group (17.9 ± 13.8 μg/mL) was higher than that in the NDM group (6.4 ± 2.0 μg/mL), and the level in the DM w/DFU group (25.9 ± 14 μg/mL) was higher than that in the DM w/o DFU group (12.3 ± 10.6 μg/mL) (Fig. 1a). Furthermore, there were more monocytes in the peripheral blood of the DM group (5.0 ± 2.1 × 10^9^/L) than in the peripheral blood of the NDM group (3.8 ± 1.1 × 10^9^/L), and the number of monocytes was much higher in the DM w/DFU group (6.2 ± 2.1 × 10^9^/L) than in the DM w/o DFU (4.1 ± 1.6 × 10^9^/L) and NDM groups (Fig. 1b). The collected data refer to the clinical data detected by a blood cell analyser. Correlation analysis indicated that circulating KBP levels were positively associated with the number of circulating monocytes in the patients in all groups (Fig. 1c, R = 0.48, *P* < 0.01). Collectively, circulating KBP levels were elevated in the DM group, especially in the DM w/DFU group, which was associated with elevated numbers of circulating monocytes.

### KBP delays wound healing, and the administration of KBP-neutralizing antibody improves wound healing in diabetic mice

**Fig. 2:**
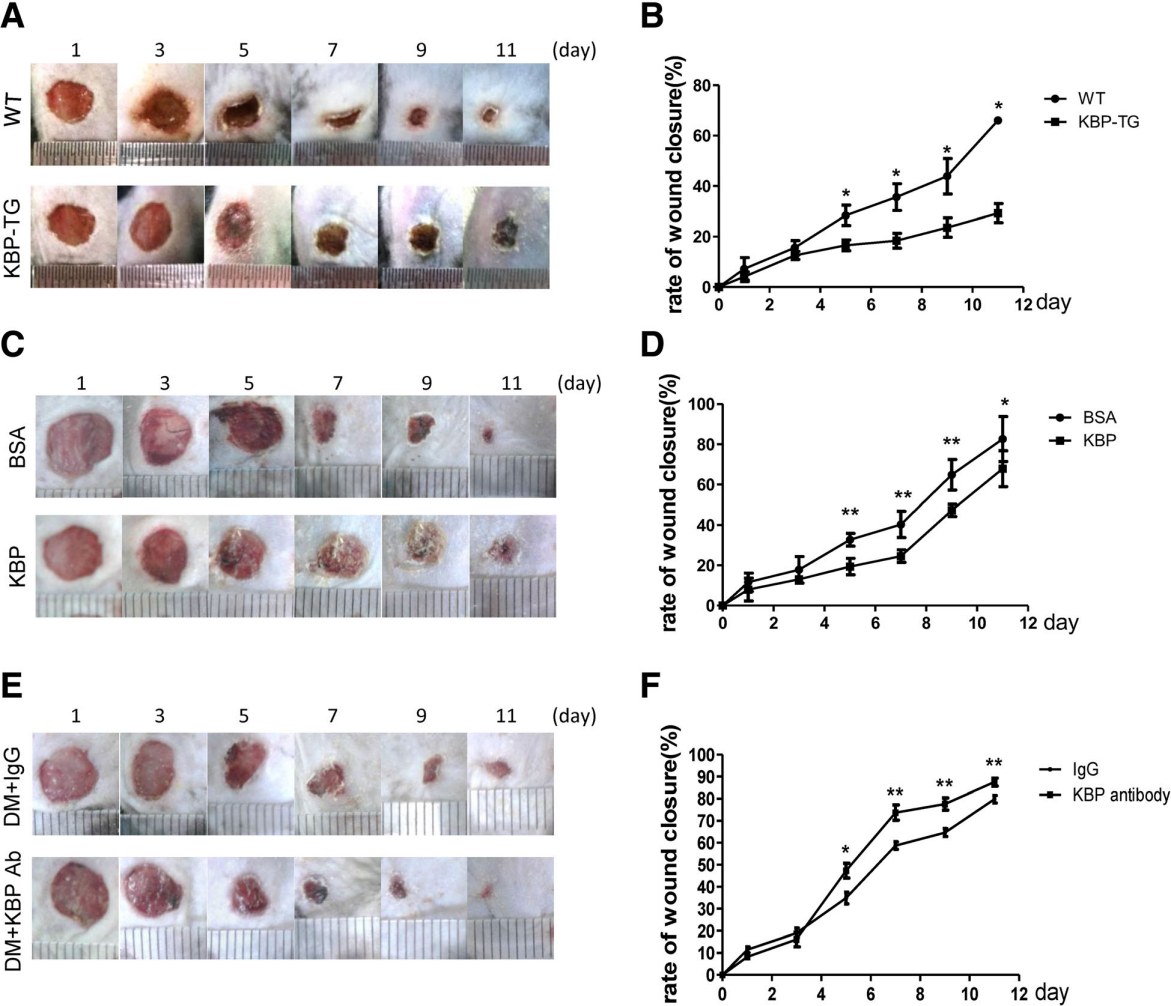
The role of KBP in wound healing. **a**, **b** Representative images showing wound healing and the wound closure rates in KBP-TG and WT mice. **c**, **d** Representative images showing wound healing and the wound closure rates in KBP-treated and BSA-treated mice. **e**, **f** Representative images showing wound healing and the wound closure rates in KBP antibody-treated type 2 diabetic mice and IgG-treated type 2 diabetic mice. Data are presented as the mean ± SD. *n* = 5; * *p* < 0.05, **p < 0.01

Wound healing in KBP-TG mice was delayed compared with that observed in wild type (WT) littermates (Fig. 2a, b). Consistently, wound healing in the recombinant KBP-treated group was slower than that in the control group treated with BSA (Fig. 2c, d). Furthermore, the administration of KBP-neutralizing antibody accelerated wound healing in diabetic mice (Fig. 2e, f) whose KBP level was elevated (Additional file 2: Figure S2). Taken together, our results suggested that KBP administration alone impaired wound healing, while wound healing in diabetic mice was accelerated via blocking KBP.

### KBP increases the number of circulating monocytes and macrophage infiltration in wounds

**Fig. 3:**
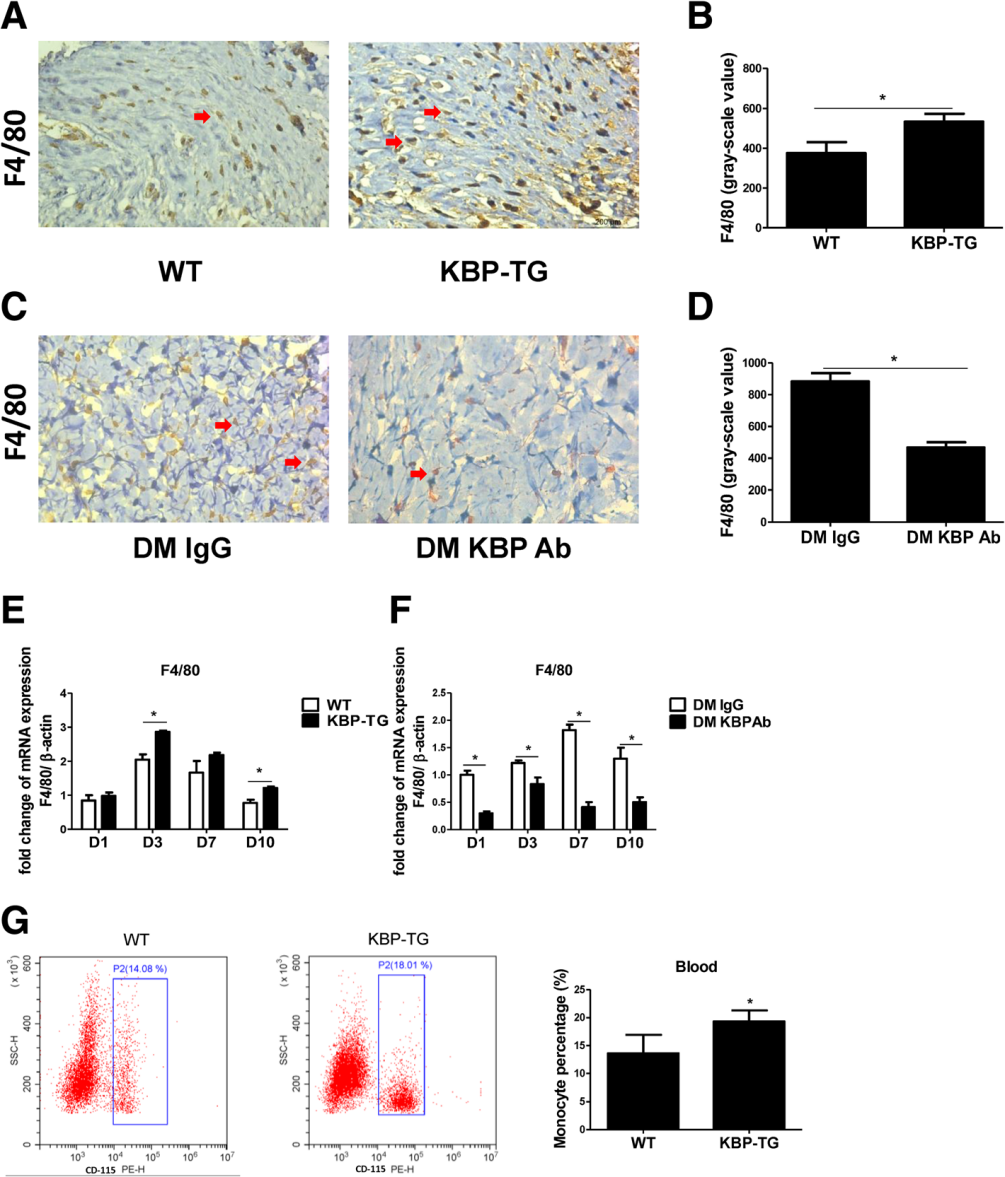
KBP increases monocyte counts in the blood and macrophage accumulation in wounds. **a**, **b** Representative immunohistochemical results and greyscale analysis of F4/80 (a macrophage marker) in the wounds of WT/KBP-TG mice at D10. **c**, **d** Representative immunohistochemical results and greyscale analysis of F4/80 in the wounds of diabetic mice treated with IgG/KBP antibody at D10. **e** The mRNA expression of F4/80 in the wounds of WT/KBP-TG mice at different time points. **f** The mRNA expression of F4/80 in the wounds of diabetic mice treated with IgG/KBP antibody at different time points. **g** Representative FACS results and the quantification of CD115^+^ monocytes in the peripheral blood of WT/KBP-TG mice. Data are presented as the mean ± SD. *n* = 3; * *p* < 0.05

Compared with that in the WT mice, there was more F4/80^+^ (a macrophage marker) macrophage infiltration in the wounds of KBP-TG mice (Fig. 3a, b), and the mRNA expression of F4/80 was correspondingly increased in the wounds of KBP-TG mice at different time points (Fig. 3e). The administration of KBP antibody decreased the infiltration of macrophages (Fig. 3c, d) as well as the mRNA expression of F4/80 (Fig. 3f). The statistical analysis of the data, which was indicated by greyscale values, is shown in Fig. 3b and Fig. 3d. In addition, the percentage of circulating monocytes was increased in KBP-TG mice (Fig. 3g) as well as db/db diabetic mice (Additional file 3: Figure S3) compared to that in control mice. Therefore, our results indicated that KBP increased the number of circulating monocytes and macrophage infiltration in wounds.

### KBP promotes the M1 polarization of macrophages

**Fig. 4:**
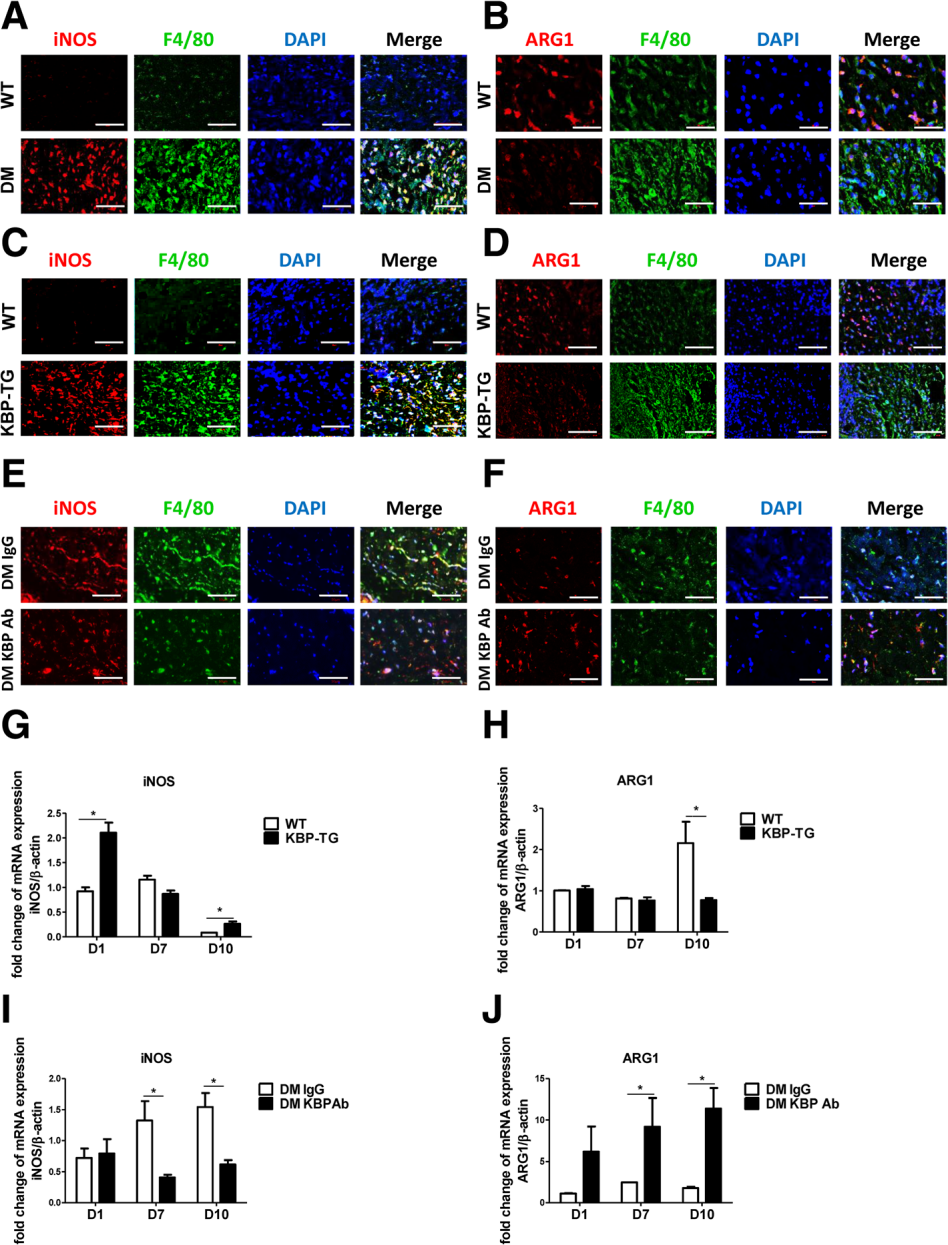
KBP promotes the M1 polarization of macrophages in vivo**. a**, **b** Representative images showing immunofluorescent staining of wound tissue sections of WT and DM mice at D10. **c**, **d** Representative images showing immunofluorescent staining of wound tissue sections of WT and KBP-TG mice at D10. **e**, **f** Representative images showing immunofluorescent staining of wound tissue sections of DM mice treated with IgG or KBP antibody at D10. (A-F) *Scale bar* = 50 μm. F4/80: macrophage marker, iNOS: M1 marker, ARG1: M2 marker. **g**, **h** The mRNA expression of iNOS and ARG1 in the wounds of WT and KBP-TG mice at different time points. **i**, **j** The expression of iNOS and ARG1 in the wounds of diabetic mice treated with IgG or KBP antibody. Data are presented as the mean ± SD. *n* = 3; * *p* < 0.05

We further examined the expression of an M1 marker (iNOS) and an M2 marker (ARG1) in vivo. There were more iNOS and F4/80 double-positive cells (Fig. 4a, c) and fewer ARG1 and F4/80 double-positive cells (Fig. 4b, d) in the wounds of DM and KBP-TG mice than those in the wounds of control mice. In contrast, the KBP antibody reduced the number of M1 macrophages and increased M2 macrophage infiltration in the wounds of diabetic mice (Fig. 4e, f). In the late stage of wound healing (D10), the mRNA expression of iNOS was increased (Fig. 4g), while the expression of ARG1 was decreased in KBP-TG mice (Fig. 4h). Similarly, treatment with KBP-neutralizing antibody reversed this phenomenon, as the expression of iNOS was downregulated (Fig. 4i), while the expression of ARG1 was upregulated in diabetic mouse wounds (Fig. 4j). Consequently, KBP increased the number of pro-inflammatory M1 macrophages and reduced M2 macrophage infiltration in diabetic wounds, especially in the late stage of wound healing, resulting in a persistent inflammatory state in the diabetic wounds.

**Fig. 5:**
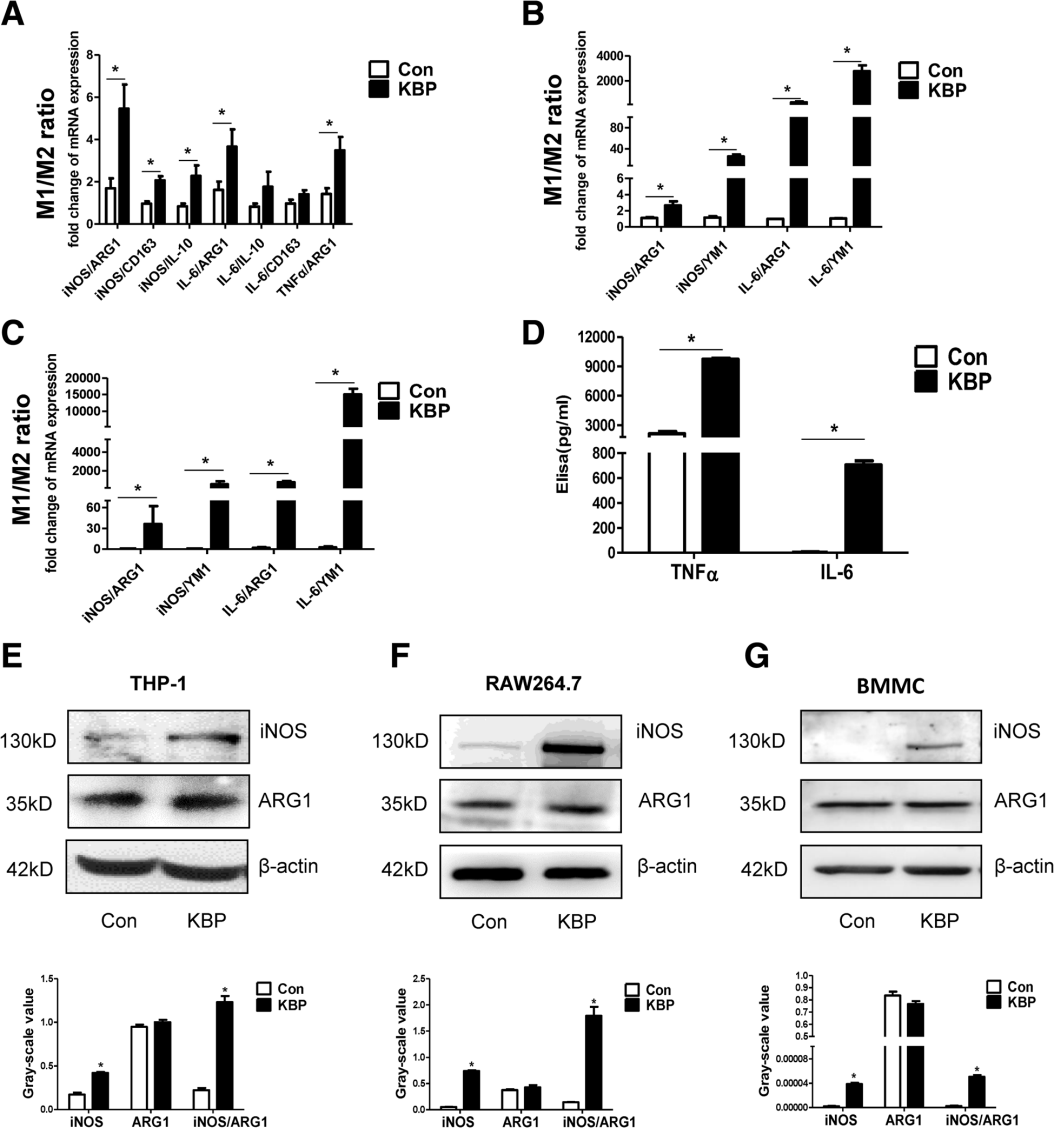
KBP stimulates M1 polarization in vitro. The mRNA expression levels of different markers/cytokines of M1 or M2 macrophages were measured, and the M1/M2 ratio was calculated. **a** The M1/M2 ratio in THP-1 cells. **b** The M1/M2 ratios of different groups in RAW264.7 cells. **c** The M1/M2 ratios of different groups in BMDMs. **d** The levels of type-1 immune cytokines (TNFα and IL-6) in the supernatants of different groups of RAW264.7 cells. **e**-**g** Western blot to detect iNOS and ARG1 in THP-1 cells, RAW264.7 cells and BMDMs together with a greyscale histogram. Data are presented as the mean ± SD. *n* = 3; * *p* < 0.05

To further explore the role of KBP in macrophage polarization, we measured different M1/M2 markers/cytokines in diverse monocyte-macrophages (Additional file 4: Figure S4A-C). The M1/M2 ratios in THP-1 cells (Fig. 5a), RAW264.7 cells (Fig. 5b) and BMDMs (Fig. 5c) stimulated with KBP were significantly increased compared to those in control cells. The expression of the inflammatory cytokines TNFα and IL-6 was also increased in the supernatant of RAW264.7 cells stimulated with KBP compared to that in unstimulated cells (Fig. 5d). The results of western blotting indicated the increased protein level of iNOS, while the expression of ARG1 was decreased or not significantly changed in the abovementioned monocyte-macrophage cell lines (Fig. 5e-g). Collectively, these results indicate that KBP stimulated the M1 polarization of macrophages both in vivo and in vitro.

### KBP promotes M1 polarization via activating the notch Signalling pathway

**Fig. 6:**
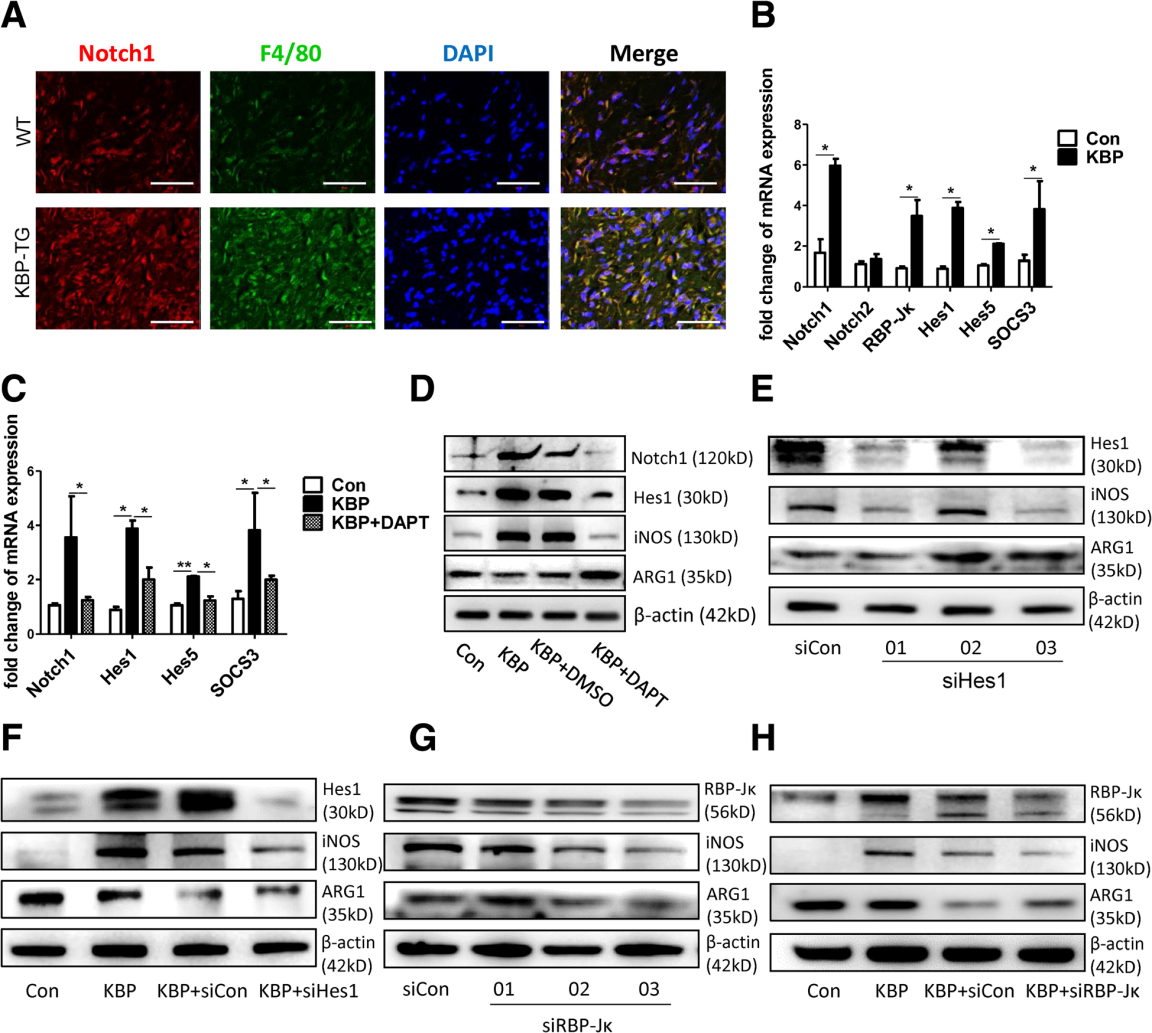
KBP promotes the M1 polarization of macrophages via activating the Notch signalling pathway. **a** The expression of Notch1 in the wounds of WT and KBP-TG mice. Scale bar = 50 μm. **b** The mRNA expression of Notch1, Notch2 and other transcription factors (RBP-Jκ) or target genes associated with Notch signalling in RAW264.7 cells. **c** DAPT inhibits the effect of KBP on the activation of Notch signalling in RAW264.7 cells. **d** Western blot to detect Notch1, Hes1, iNOS and ARG1 following the treatment of RAW264.7 cells with KBP and DAPT. **e**, **g** The expression of iNOS and ARG1 in RAW264.7 cells following treatment with siHes1 and siRBP-Jκ. (F, H) The expression of iNOS and ARG1 in RAW264.7 cells following treatment with KBP and siHes1 or siRBP-Jκ. Data are presented as the mean ± SD. *n* = 3; * *p* < 0.05. Three independent experiments were employed

Further, we explored the effect of KBP on macrophage M1 polarization. The expression of Notch1 was upregulated in the wound bed of KBP-TG mice compared with that in WT mice (Fig. 6a). In RAW264.7 cells, the mRNA expression of Notch1 and its downstream transcription factor or target genes (such as *RBP-Jκ, Hes1, Hes5 and Socs3*) were increased after KBP treatment (Fig. 6b). DAPT, a Notch signalling inhibitor, repressed the upregulation of Notch1, Hes1, Hes5 and SOCS3 following treatment with KBP (Fig. 6c). The results of immunofluorescent staining of Notch1 were consistent with those above (Additional file 5: Figure S5A), and the expression of delta-like 4 (DLL4, a Notch receptor) was also increased after KBP treatment in RAW264.7 macrophages (Additional file 5: Figure S5B). In addition, DAPT inhibited the expression of iNOS and upregulated the expression of ARG1 via inhibiting the Notch signalling pathway following treatment with recombinant KBP (Fig. 6d). Therefore, inhibition of the Notch signalling pathway reversed the effect of KBP on macrophage polarization. To further explore the molecular mechanism of this effect, we employed three pairs of siRNA sequences that knocked down RBP-Jκ or Hes1, reduced the expression of iNOS and increased ARG1 compared with that in the control in RAW264.7 macrophages (Fig. 6e, g). In addition, macrophages were treated with KBP and siRBP-Jκ 03 or siHes1 03, which was most effective, and the effect of KBP on macrophage polarization was reversed following the interference of RBP-Jκ and Hes1 (Fig. 6f, h). Our observations indicated that KBP promoted the M1 polarization of macrophages through activating the Notch signalling pathway.

### Hes1, a downstream target gene of the notch Signalling pathway, does not directly activate iNOS expression

**Fig. 7:**
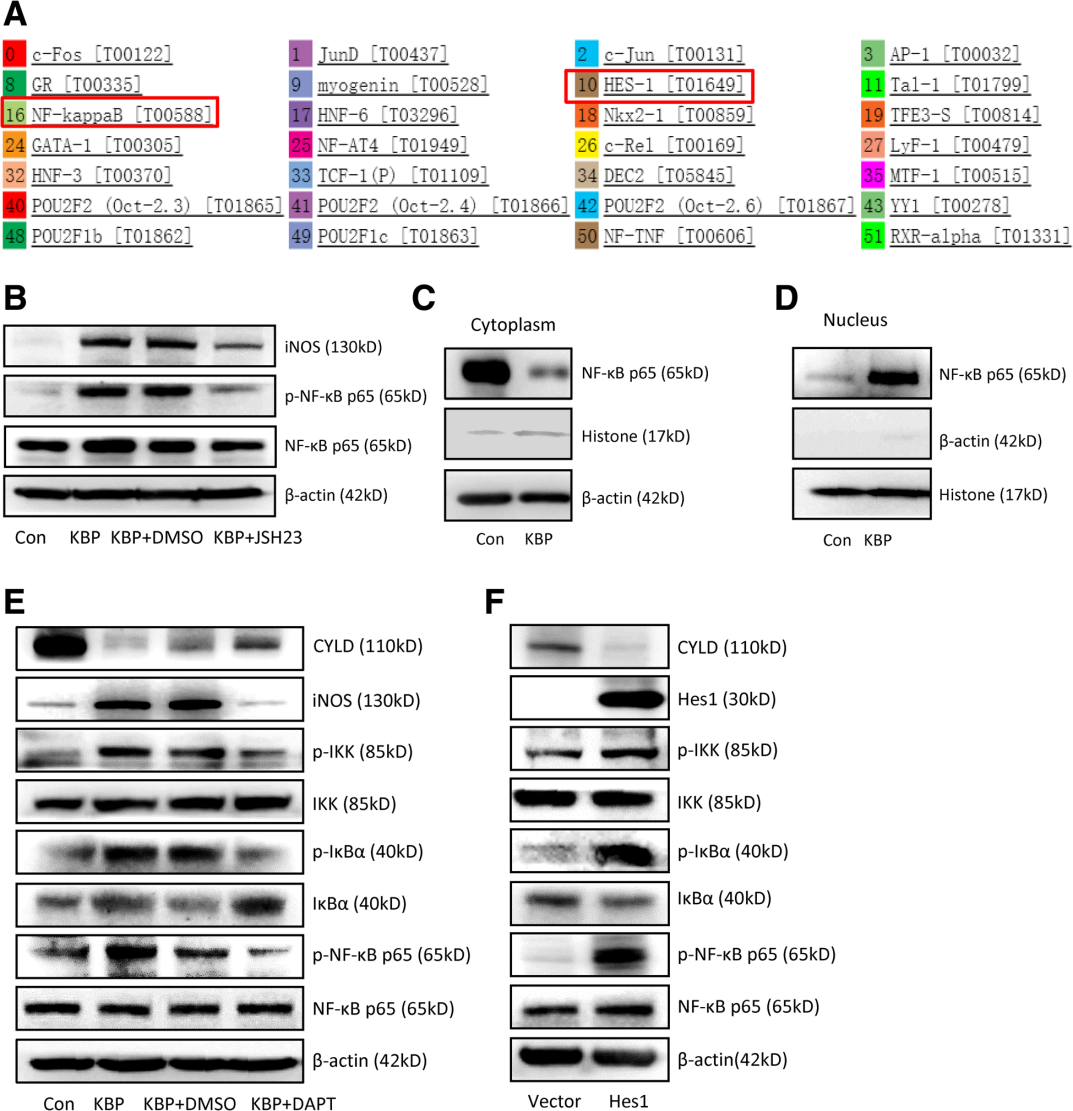
KBP stimulates the M1 polarization of macrophages via cross-activation of the Notch and NF-κB signalling pathways. **a** Bioinformatics prediction via the PROMO website of the possible transcription factors that bind to the iNOS promoter region. **b** The expression of iNOS and molecules in the NF-κB signalling pathway in RAW264.7 cells treated with KBP with or without JSH23. **c** The expression of NF-κB p65 in the cytoplasm of RAW264.7 cells. **d** The expression of NF-κB p65 in the nuclei of RAW264.7 cells. **e** The expression of CYLD, iNOS and molecules in the NF-κB signalling pathway in RAW264.7 cells treated with KBP with or without DAPT. **f** The expression of CYLD, iNOS and molecules in the NF-κB signalling pathway in RAW264.7 cells transfected with Hes1 or control vector. Three independent experiments were employed

Furthermore, we used a bioinformatics method to predict the possible transcription factors that bind to the promoter region of iNOS via the PROMO website. Bioinformatics prediction indicated that Hes1, a downstream target gene of Notch signalling, could bind to the promoter region of the iNOS gene (Fig. 7a). The mRNA expression of iNOS was decreased after the interference of Hes1 (Additional file 6: Figure S6A). A ChIP assay showed that Hes1 bound to the promoter region of the iNOS gene (Additional file 6: Figure S6B), while a dual-luciferase reporter gene assay indicated that the transcription of iNOS was not activated by Hes1 in 293 T cells (Additional file 6: Figure S6C). Our observation indicated that Hes1 bound the promoter region of the iNOS gene but did not activate the expression of iNOS directly.

### KBP promotes the M1 polarization of macrophages via activation of the notch Signalling pathway and the cross-activation of the NF-κB inflammatory Signalling pathway

NF-κB is a classical inflammatory signalling pathway that promotes the M1 polarization of macrophages, and NF-κB is also a possible transcription factor that binds to the promoter region of iNOS (Fig. 7a). The activation of Notch signalling activated the NF-κB signalling pathway in breast cancer cells, and NF-κB activated the transcription of iNOS directly [28]. Our results indicated that KBP promoted the phosphorylation and activation of NF-κB p65 (Fig. 7b, e) as well as the translocation of p65 into the nucleus (Fig. 7c, d). Furthermore, KBP promoted the phosphorylation of inhibitor of κB (IκBα) kinase (IKK) to activate the phosphorylation of downstream IκBα, which inhibits the NF-κB transcription factor and is inactive in its phosphorylated form (Fig. 7e). In addition, KBP downregulated the expression of cylindromatosis tumour-suppressor protein (CYLD) (Fig. 7e), which is a deubiquitinase and negative regulator of NF-κB signalling [39]. Treatment with the NF-κB signalling inhibitor JSH23 and the Notch signalling inhibitor DAPT inhibited the effect of KBP on the activation of the NF-κB signalling pathway (Fig. 7b-e), while the overexpression of Hes1 activated the NF-κB signalling pathway (Fig. 7f). The above results indicated that KBP promoted the M1 polarization of macrophages via activation of Notch signalling and cross-activation of the NF-κB signalling pathway.

### KBP upregulates the expression of M-CSF and MCP-1

**Fig. 8:**
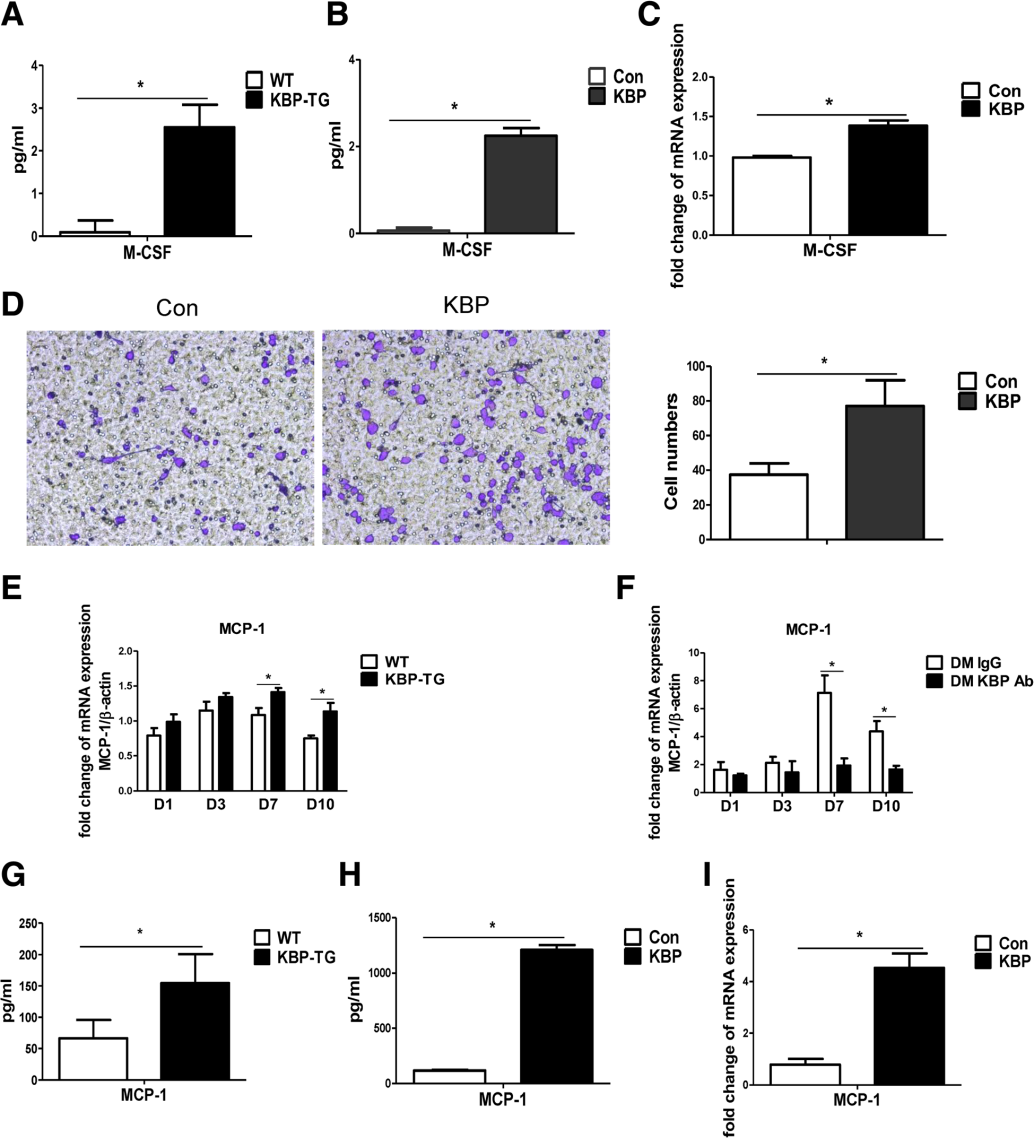
KBP increases the production of M-CSF and MCP-1, which employs the differentiation and migration of monocytes and macrophages. **a** The plasma level of M-CSF in WT and KBP-TG mice. **b** The level of M-CSF in the cell supernatant of RAW 264.7 cells. **c** The mRNA expression of M-CSF in RAW264.7 cells treated with KBP versus that in the Con group. **d** KBP stimulates the migration of macrophages. Representative images of the Transwell migration assay and a statistical histogram. **e** The mRNA expression of MCP-1 in the wounds of WT and KBP-TG mice at different time points. **f** The mRNA expression of MCP-1 in the wounds of DM mice treated with IgG and KBP antibody at different time points. **g** The plasma level of MCP-1 in WT and KBP-TG mice. **h** The level of MCP-1 in the cell supernatant of RAW264.7 cells treated with KBP versus that in the Con group. The supernatant of RAW264.7 cells treated with KBP versus that of the Con group. I. The mRNA expression of MCP-1 in RAW264.7 cells treated with KBP versus that in the Con group. Data are presented as the mean ± SD. *n* = 3; * *p* < 0.05

M-CSF was upregulated in the plasma of KBP-TG mice (Fig. 8a) as well as the supernatant of RAW264.7 macrophages treated with KBP (Fig. 8b). KBP increased M-CSF mRNA expression in RAW264.7 cells (Fig. 8c). In a Transwell migration assay, RAW264.7 cells treated with KBP exhibited increased migration compared to that of the control group (Fig. 8d), which may be associated with the upregulation of MCP-1. Furthermore, the increased expression of MCP-1 was detected in the wounds of KBP-TG mice, especially in the late stage of wound healing, while KBP antibody reversed this phenomenon (Fig. 8e, f). MCP-1 was upregulated in the plasma of KBP-TG mice (Fig. 8g) and the supernatant of the RAW264.7 cells (Fig. 8h), and the mRNA expression of MCP-1 was correspondingly increased (Fig. 8i). Consequently, our results indicated that KBP promoted the differentiation and chemotactic migration of monocytes-macrophages via the upregulation of M-CSF and MCP-1.

## Discussion

An excessive inflammatory reaction delays diabetic healing, which is a conjoint cause of amputation in diabetic patients [16, 40]. The molecular basis underlying the pathogenesis of excessive inflammatory reactions in diabetes-induced wound healing deficiency has not been completely illuminated. The present study demonstrated an association between elevated levels of circulating KBP and increased numbers of monocyte-macrophages in DFU for the first time. Furthermore, circulating monocyte-macrophages and macrophage infiltration were upregulated in KBP-TG mice compared to those in control mice. We have demonstrated that 1) high levels of KBP contributed to a delay in wound healing in diabetic mice through regulating monocyte-macrophages that induced an excessive inflammatory reaction and that 2) KBP promoted the M1 polarization of macrophages, resulting in the accumulation of pro-inflammatory M1 macrophages and a prolonged inflammatory state. Our studies suggest for the first time that KBP may promote M1 polarization through activating the Notch and NF-κB signalling pathways and that Hes1 may activate the NF-κB signalling pathway via inhibiting CYLD. These observations established for the first time an association between elevated KBP levels and an excessive inflammatory reaction with delayed diabetic healing and DFU, which may provide a new theoretical basis for and targets to intervene in DFU.

Our previous studies demonstrated that circulating KBP levels were increased in diabetic patients associated with microvascular complications [20, 21]. Here, we revealed that circulating KBP levels were elevated in DM patients, especially DM w/DFU patients, compared to those in nondiabetic individuals, which was associated with elevated monocyte counts (Fig. 1). Furthermore, our results revealed delayed wound healing in KBP-TG mice and recombinant KBP-treated mice compared with that in WT littermates (Fig. 2a-d). This is authoritative evidence suggesting that KBP may be a factor in the regulation of wound healing. To further establish the role of KBP in wound healing, the administration of a KBP-neutralizing antibody was used to block KBP activity, which accelerated wound healing in diabetic mice (Fig. 2e, f). Taken together, these results all suggest that elevated levels of circulating KBP indeed contribute to a delay in wound healing in diabetes.

Diabetic patients have delayed healing characterized by persistent inflammatory responses accompanied by the prolonged accumulation of M1 macrophages, which can eventually necessitate lower limb amputation [16]. Elevated KBP was associated with an increased number of circulating monocytes in diabetic patients with DFU compared with those in diabetic patients without DFU. Therefore, we hypothesized that a high level of KBP might influence persistent inflammatory responses in diabetes through regulating the recruitment and polarization of macrophages. Consistent with this prediction, our study demonstrated that the number of circulating monocytes and macrophage infiltration in the wound were increased in KBP-TG mice compared to those in control mice (Fig. 3a, b, e, g). KBP administration promoted the recruitment of macrophages and M1 polarization in an animal model and various monocyte-macrophage cell lines (Figs. 4 and 5), which suggesting KBP induced the persistent inflammatory responses in diabetic wound tissue. However, previous studies have suggested that KBP has potent anti-inflammatory activities: such as Liu’s study demonstrated KBP decreased inflammatory cell infiltration and TNFα express in the corneal, which represent a superficial angiogenesis and acute inflammation model [41]. While chronic inflammation is a hallmark of impaired diabetic wound healing [42]. These results suggested that KBP may play the diverse roles in different patterns of inflammation. Our results confirmed that the polarization and recruitment of macrophages are crucial in the inflammatory response during wound healing [10–12]. Nevertheless, the underlying molecular mechanism is not well understood.

Notch signalling plays a pivotal role in regulating the development and differentiation of monocyte-macrophages [23, 24]. Increased M1 macrophage infiltration was correlated with the activation of Notch signalling in the wounds of KBP-TG mice (Fig. 6A). To further confirm that Notch signalling contributes to macrophage polarization, DAPT, an inhibitor of the Notch pathway, and knockdown of the transcription factor RBP-Jκ and Hes1 by siRNA were employed to explore the effects of KBP on Notch signalling. DAPT downregulated the expression of iNOS and upregulated the expression of ARG1 via inhibiting the Notch signalling pathway under KBP treatment (Fig. 6b-h). Taken together, our observations indicate for the first time that KBP promoted the M1 polarization of macrophages through activating the Notch signalling pathway.

Bioinformatics prediction and a ChIP assay showed that Hes1 could bind to the promoter of iNOS, while a dual-luciferase reporter gene assay showed that Hes1 could not activate the expression of iNOS directly (Additional file 6: Figure S6). Since Hes1 could not activate iNOS expression directly, we wondered whether KBP activated iNOS expression via an indirect pathway. The NF-κB signalling pathway, which is a classic pathway that promotes the M1 polarization of macrophages, is closely related to the inflammatory response [13, 43]. Hes1, which is downstream of Notch signalling, can inhibit the transcription of the deubiquitinase CYLD, which negatively regulates IKK [44]. CYLD inhibits the ubiquitination of TNFα receptor-associated factor (TRAF6), while TRAF6 conjugated to a Lys-63 (K63)-linked polyubiquitin chain is required for the activation of IKK and downstream signalling events [45–47]. KBP activated the Notch signalling pathway to upregulate Hes1, which inhibited the expression of CYLD to activate the phosphorylation of IKK in macrophages. After the activation of IKK, NF-κB signalling was activated, and the subsequent nuclear translocation of p65 further promotes iNOS expression, which keeps macrophages in an M1 polarization state (Fig. 7e, f). We first discovered the effect of KBP in promoting the M1 polarization of macrophages by cross-activating the Notch pathway and NF-κB signalling pathways (Additional file 7: Figure S1). A similar mechanism was also found in breast cancer cells [28].

Macrophages come from monocytes and upstream progenitor cells that are regulated by M-CSF [5]. In wounded tissue, macrophage recruitment depended on the ischaemia-induced upregulation of MCP-1 and the increased expression of CCR2 on the cell surface. We also explored the possible mechanism by which KBP regulates monocyte-macrophage numbers. Since the differentiation, mobilization and recruitment of macrophages are regulated by M-CSF and MCP-1, we found the increased expression of M-CSF and MCP-1 following treatment with recombinant KBP in KBP-TG mice (Fig. 8). The detailed mechanism by which KBP regulates M-CSF and MCP-1 remains to be clarified in the future.

## Conclusions

As shown by these results, KBP aggravated the inflammatory response in wound tissue by targeting macrophages. We first demonstrated that a high level of KBP in DFUs activated Notch signalling and the NF-κB signalling pathway, leading to M1 polarization, increased numbers of macrophages in the wound, and, consequently, excessive inflammatory reactions during wound healing. These activities contribute to a delay in wound healing in diabetic patients. Hence, the blockade of KBP may benefit DFU treatment and prevent amputation.

## Additional files

Additional file 1: **Table S1.** Clinical characteristics in non-diabetic (NDM) control subjects, patients with Type 2 diabetes (DM) and diabetic patients with diabetic foot ulcer (DM + DFU). **Table S2.** Primer sequences for real-time quantitative PCR. (DOC 65 kb)

Additional file 2: **Figure S2.** The expression of KBP in the liver tissue of WT and diabetic mice (*n* = 3). (DOCX 214 kb) (TIFF 1695 kb)

Additional file 3: **Figure S3.** Representative FACS results and quantification of CD115^+^ monocytes in peripheral blood of WT and db/db male mice. All data were presented as the mean ± SD. *n* = 3; * *p* < 0.05. (TIFF 2174 kb)

Additional file 4: **Figure S4.** A. The mRNA expression of M1 and M2 markers/cytokines in THP-1 cells. B. The mRNA expression of M1 and M2 markers/cytokines in RAW264.7 cells. C. The mRNA expression of M1 and M2 markers/cytokines in BMDM. Data are presented as the mean ± SD. n = 3; * *p* < 0.05. (TIFF 22 kb)

Additional file 5: **Figure S5.** A. The immunofluorescence staining results of notch1 in different groups of RAW264.7 macrophages. *Scale bar* = 5 μm. B. The expression of Jagged1 and DLL4 in RAW264.7 cells. (TIFF 4456 kb)

Additional file 6: **Figure S6.** A. The mRNA expression of iNOS in RAW264.7 cells after using sihes1. Data were presented as the mean ± SD. n = 3; * *p* < 0.05 versus sicon group. B. The ChIP assay of transcription factor hes1 and iNOS promoter region. C. The dual-luciferase report gene assay for hes1 and iNOS promoter in 293 T cells. (TIFF 1669 kb)

Additional file 7: **Figure S1.** The schematic overview of KBP in promoting the M1 polarization of macrophages by cross-activating the notch signaling pathway and NF-κB signaling pathway. KBP upregulates DLL4 and Notch1 to activate notch signaling, promoting NICD to bind to the CSL/RBPJ-κ, and then increases the expression of hes1. Hes1 suppresses the expression of CYLD, which could inhibit the ubiquitination of TRAF6. Because of the reduction of CYLD, the weakened deubiquitination of TRAF6 could activate NF-κB p65 via phosphorylation of IκBα. NF-κB p65 translocases into the nucleus to activate the expression of target gene-iNOS, to promote the M1 polarization of macrophages. (TIFF 2700 kb)

## Data Availability

All data generated or analysed during this study are included in this published article and its supplementary information files.

## Abbreviations

ARG1: Arginase-1
CCR2: Chemokine receptor 2
DFU: Diabetic foot ulcer
DM w/DFU: Diabetic patients with diabetic foot ulcer
DM w/o DFU: Diabetic patients without diabetic foot ulcer
IKK: Inhibitor of κB kinase
iNOS: Nitric oxide synthase
IκBα: Inhibitor of κB
KBP: Kallikrein-binding protein
MCP-1: Monocyte chemoattractant protein-1
M-CSF: Macrophage colony-stimulating factor
NDM: Nondiabetic control
NICD: Notch intracellular domain

## References

[1] Boulton AJ, Vileikyte L, Ragnarson-Tennvall G, Apelqvist J (2005). The global burden of diabetic foot disease. Lancet.

[2] White R, McIntosh C (2009). A review of the literature on topical therapies for diabetic foot ulcers. Part 2: advanced treatments. J Wound Care.

[3] Singer AJ, Clark RA (1999). Cutaneous wound healing. N Engl J Med.

[4] Qi W, Yang C, Dai Z, Che D, Feng J, Mao Y, Cheng R, Wang Z, He X, Zhou T (2015). High levels of pigment epithelium-derived factor in diabetes impair wound healing through suppression of Wnt signaling. Diabetes.

[5] Bennett CL, Djulbegovic B, Norris LB, Armitage JO (2013). Colony-stimulating factors for febrile neutropenia during cancer therapy. N Engl J Med.

[6] Ushach I, Zlotnik A (2016). Biological role of granulocyte macrophage colony-stimulating factor (GM-CSF) and macrophage colony-stimulating factor (M-CSF) on cells of the myeloid lineage. J Leukoc Biol.

[7] Lee HW, Choi HJ, Ha SJ, Lee KT, Kwon YG (2013). Recruitment of monocytes/macrophages in different tumor microenvironments. Biochim Biophys Acta.

[8] Kolattukudy PE, Niu J (2012). Inflammation, endoplasmic reticulum stress, autophagy, and the monocyte chemoattractant protein-1/CCR2 pathway. Circ Res.

[9] Daley JM, Brancato SK, Thomay AA, Reichner JS, Albina JE (2010). The phenotype of murine wound macrophages. J Leukoc Biol.

[10] Porcheray F, Viaud S, Rimaniol AC, Leone C, Samah B, Dereuddre-Bosquet N, Dormont D, Gras G (2005). Macrophage activation switching: an asset for the resolution of inflammation. Clin Exp Immunol.

[11] Gordon S (2003). Alternative activation of macrophages. Nat Rev Immunol.

[12] Mosser DM, Edwards JP (2008). Exploring the full spectrum of macrophage activation. Nat Rev Immunol.

[13] Sica A, Mantovani A (2012). Macrophage plasticity and polarization: in vivo veritas. J Clin Invest.

[14] Brancato SK, Albina JE (2011). Wound macrophages as key regulators of repair Origin, Phenotype, and Function. Am J Pathol.

[15] Goren I, Allmann N, Yogev N, Schurmann C, Linke A, Holdener M, Waisman A, Pfeilschifter J, Frank S (2009). A transgenic mouse model of inducible macrophage depletion: effects of diphtheria toxin-driven lysozyme M-specific cell lineage ablation on wound inflammatory, angiogenic, and contractive processes. Am J Pathol.

[16] Loots MA, Lamme EN, Zeegelaar J, Mekkes JR, Bos JD, Middelkoop E (1998). Differences in cellular infiltrate and extracellular matrix of chronic diabetic and venous ulcers versus acute wounds. J Invest Dermatol.

[17] Chai KX, Ma JX, Murray SR, Chao J, Chao L (1991). Molecular cloning and analysis of the rat kallikrein-binding protein gene. J Biol Chem.

[18] Miao RQ, Agata J, Chao L, Chao J (2002). Kallistatin is a new inhibitor of angiogenesis and tumor growth. Blood.

[19] Shen B, Hagiwara M, Yao YY, Chao L, Chao J (2008). Salutary effect of kallistatin in salt-induced renal injury, inflammation, and fibrosis via antioxidative stress. Hypertension.

[20] Jenkins AJ, McBride JD, Januszewski AS, Karschimkus CS, Zhang B, O'Neal DN, Nelson CL, Chung JS, Harper CA, Lyons TJ, Ma JX (2010). Increased serum kallistatin levels in type 1 diabetes patients with vascular complications. J Angiogenes Res.

[21] McBride JD, Jenkins AJ, Liu XC, Zhang B, Lee K, Berry WL, Janknecht R, Griffin CT, Aston CE, Lyons TJ (2014). Elevated circulation levels of an antiangiogenic SERPIN in patients with diabetic microvascular complications impair wound healing through suppression of Wnt signaling. J Investig Dermatol.

[22] Kopan R, Ilagan MX (2009). The canonical notch signaling pathway: unfolding the activation mechanism. Cell.

[23] Xu H, Zhu J, Smith S, Foldi J, Zhao B, Chung AY, Outtz H, Kitajewski J, Shi C, Weber S (2012). Notch-RBP-J signaling regulates the transcription factor IRF8 to promote inflammatory macrophage polarization. Nat Immunol.

[24] Wang YC, He F, Feng F, Liu XW, Dong GY, Qin HY, Hu XB, Zheng MH, Liang L, Feng L (2010). Notch signaling determines the M1 versus M2 polarization of macrophages in antitumor immune responses. Cancer Res.

[25] Wan F, Lenardo MJ (2010). The nuclear signaling of NF-kappaB: current knowledge, new insights, and future perspectives. Cell Res.

[26] Hacker H, Karin M (2006). Regulation and function of IKK and IKK-related kinases. Sci STKE.

[27] Gonzalez DM, Medici D (2014). Signaling mechanisms of the epithelial-mesenchymal transition. Sci Signal.

[28] Li L, Zhang J, Xiong N, Li S, Chen Y, Yang H, Wu C, Zeng H, Liu Y (2016). Notch-1 signaling activates NF-kappaB in human breast carcinoma MDA-MB-231 cells via PP2A-dependent AKT pathway. Med Oncol.

[29] Maniati E, Bossard M, Cook N, Candido JB, Emami-Shahri N, Nedospasov SA, Balkwill FR, Tuveson DA, Hagemann T (2011). Crosstalk between the canonical NF-kappaB and notch signaling pathways inhibits Ppargamma expression and promotes pancreatic cancer progression in mice. J Clin Invest.

[30] Fukuda D, Aikawa E, Swirski FK, Novobrantseva TI, Kotelianski V, Gorgun CZ, Chudnovskiy A, Yamazaki H, Croce K, Weissleder R (2012). Notch ligand delta-like 4 blockade attenuates atherosclerosis and metabolic disorders. Proc Natl Acad Sci U S A.

[31] Furman BL (2015). Streptozotocin-induced diabetic models in mice and rats. Curr Protoc Pharmacol.

[32] Yu T, Sungelo MJ, Goldberg IJ, Wang H, Eckel RH (2017). Streptozotocin-treated high fat fed mice: a new type 2 diabetes model used to study Canagliflozin-induced alterations in lipids and lipoproteins. Horm Metab Res.

[33] Yang Z, Grinchuk V, Urban JF, Bohl J, Sun R, Notari L, Yan S, Ramalingam T, Keegan AD, Wynn TA (2013). Macrophages as IL-25/IL-33-responsive cells play an important role in the induction of type 2 immunity. PLoS One.

[34] Dai Z, Qi W, Li C, Lu J, Mao Y, Yao Y, Li L, Zhang T, Hong H, Li S (2013). Dual regulation of adipose triglyceride lipase by pigment epithelium-derived factor: a novel mechanistic insight into progressive obesity. Mol Cell Endocrinol.

[35] Zhang B, Zhou KK, Ma JX (2010). Inhibition of connective tissue growth factor overexpression in diabetic retinopathy by SERPINA3K via blocking the WNT/beta-catenin pathway. Diabetes.

[36] Li S, Mao Y, Zhou T, Luo C, Xie J, Qi W, Yang Z, Ma J, Gao G, Yang X (2016). Manganese superoxide dismutase mediates anoikis resistance and tumor metastasis in nasopharyngeal carcinoma. Oncotarget.

[37] Hong H, Zhou T, Fang S, Jia M, Xu Z, Dai Z, Li C, Li S, Li L, Zhang T (2014). Pigment epithelium-derived factor (PEDF) inhibits breast cancer metastasis by down-regulating fibronectin. Breast Cancer Res Treat.

[38] Kadariya Y, Nakatani K, Nishioka J, Fujikawa T, Kruger WD, Nobori T (2005). Regulation of human methylthioadenosine phosphorylase gene by the CBF (CCAAT binding factor)/NF-Y (nuclear factor-Y). Biochem J.

[39] Hahn M, Burckert JP, Luttenberger CA, Klebow S, Hess M, Al-Maarri M, Vogt M, Reissig S, Hallek M, Wienecke-Baldacchino A, et al. Aberrant splicing of the tumor suppressor CYLD promotes the development of chronic lymphocytic leukemia via sustained NF-kappaB signaling. Leukemia. 2017. 10.1038/leu.2017.168 28566736

[40] Brem H, Tomic-Canic M (2007). Cellular and molecular basis of wound healing in diabetes. J Clin Invest.

[41] Liu Xiaochen, Lin Zhirong, Zhou Tong, Zong Ronrong, He Hui, Liu Zhen, Ma Jian-xing, Liu Zuguo, Zhou Yueping (2011). Anti-Angiogenic and Anti-Inflammatory Effects of SERPINA3K on Corneal Injury. PLoS ONE.

[42] Torbica T, Wicks K, Umehara T, Gungordu L, Alrdahe S, Wemyss K, Grainger JR, Mace KA. Chronic inflammation in response to injury: retention of myeloid cells in injured tissue is driven by myeloid cell-intrinsic factors. J Invest Dermatol. 2019. 10.1016/j.jid.2018.12.030 30703358

[43] Simon PS, Sharman SK, Lu C, Yang D, Paschall AV, Tulachan SS, Liu K (2015). The NF-kappaB p65 and p50 homodimer cooperate with IRF8 to activate iNOS transcription. BMC Cancer.

[44] Espinosa L, Cathelin S, D'Altri T, Trimarchi T, Statnikov A, Guiu J, Rodilla V, Ingles-Esteve J, Nomdedeu J, Bellosillo B (2010). The notch/Hes1 pathway sustains NF-kappaB activation through CYLD repression in T cell leukemia. Cancer Cell.

[45] Jin W, Chang M, Paul EM, Babu G, Lee AJ, Reiley W, Wright A, Zhang M, You J, Sun SC (2008). Deubiquitinating enzyme CYLD negatively regulates RANK signaling and osteoclastogenesis in mice. J Clin Invest.

[46] Deng L, Wang C, Spencer E, Yang L, Braun A, You J, Slaughter C, Pickart C, Chen ZJ (2000). Activation of the IkappaB kinase complex by TRAF6 requires a dimeric ubiquitin-conjugating enzyme complex and a unique polyubiquitin chain. Cell.

[47] Wang C, Deng L, Hong M, Akkaraju GR, Inoue J, Chen ZJ (2001). TAK1 is a ubiquitin-dependent kinase of MKK and IKK. Nature.

