# Supplementary material for: Elevated Kallikrein-binding protein in diabetes impairs wound healing through inducing macrophage M1 polarization
Source: Cell Commun Signal. 2019 Jun 10;17:60. doi: 10.1186/s12964-019-0376-9 (PMC6558923; doi:10.1186/s12964-019-0376-9)
Supplement: Supplementary file 1 — Table S1. Clinical characteristics in non-diabetic (NDM) control subjects, patients with Type 2 diabetes (DM) and diabetic patients with diabetic foot ulcer (DM + DFU). Table S2. Primer sequences for real-time quantitative PCR. (DOC 65 kb) [file 12964_2019_376_MOESM1_ESM.doc]

**Additional file 1**

Table S1. Clinical characteristics in non-diabetic (NDM) control subjects, patients with Type 2 diabetes (DM) and diabetic patients with diabetic foot ulcer (DM+DFU).

|  | NDM | | DM | | DM+DFU | | *p* value  (NDM vs DM) | | *p* value  (DM vs DM+DFU) | |
| --- | --- | --- | --- | --- | --- | --- | --- | --- | --- | --- |
| n | 61 | 44 | | 25 | | __ | | __ | |  |
| Age | 60±9 | | 59±12 | | 64±10 | | 0.75 | 0.09 | | |
| TC (mg/dl) | 4.56±1.47 | | 4.97±1.24 | | 4.75±2.0 | | 0.20 | 0.59 | | |
| LDL (mg/dl) | 2.92±1.44 | | 3.29±0.88 | | 3.18±1.59 | | 0.21 | 0.73 | | |
| HDL (mg/dl) | 1.26±0.28 | | 1.16±0.23 | | 1.11±0.39 | | 0.11 | 0.56 | | |
| TG (mg/dl) | 1.23±0.6 | | 1.61±1.38 | | 1.58±0.79 | | 0.14 | 0.93 | | |
| PLT (×10^9^/L) | 253.72±74.75 | | 241.63±66.17 | | 321.52±77.26 | | 0.46 | 0.00 | | |
| WBC (×10^9^/L) | 6.59±1.99 | | 7.21±2.20 | | 9.10±2.48 | | 0.22 | 0.00 | | |
| RBC (×10^12^/L) | 13.32±42.66 | | 4.48±0.80 | | 3.93±0.63 | | 0.2 | 0.01 | | |
| Hb (g/L) | 130.41±26.99 | | 129.08±22.06 | | 113.80±19.62 | | 0.83 | 0.01 | | |
| UA (μmol/L) | 344.78±111.60 | | 339.17±135.15 | | 373.3±169.91 | | 0.27 | 0.40 | | |
| GLU (mmol/L) | 5.01±0.45 | | 11.74±5.43 | | 13.32±7.40 | | 0.00 | 0.39 | | |

Table S2. Primer sequences for real-time quantitative PCR.

Gene Primer sequences (5’ to 3’)

*mF4/80* Forward, CCTGGACGAATCCTGTGAAG

Reverse, GGTGGGACCACAGAGAGTTG

*miNOS* Forward, CGGAGCCTTTAGACCTCAACA

Reverse, CCCTCGAAGGTGAGCTGAAC

*mARG1* Forward, CTGGCAGTTGGAAGCATCTCT

Reverse, GTGAGCATCCACCCAAATGAC

*mIL-6* Forward, TCCATCCAGTTGCCTTCTTG

Reverse, TTTCTCATTTCCACGATTTCCC

*mYM-1* Forward, ATCTATGCCTTTGCTGGAATGC

Reverse, TGAATGAATATCTGACGGTTCTGAG

*hiNOS* Forward, CCTTACGAGGCGAAGAAGGAC

Reverse, CAGTTTGAGAGAGGAGGCTCCG

*hIL-6* Forward, TCCTGCAGAAAAAGGCAAAGAATC

Reverse, GCGCAGAATGAGATGAGTTGT

*hARG1* Forward, CAAGAAGAACGGAAGAATCAGC

Reverse, TTGTGGTTGTCAGTGGAGTGTT

*hCD163* Forward, TTGCCAGCAGTTAAATGTG

Reverse, AGGACAGTGTTTGGGACTGG

*hIL-10* Forward, GATCCAGTTTTACCTGGAGGA

Reverse, CCTGAGGGTCTTCAGGTTCTC

*hTNFα* Forward, CGAGTGACAAGCCTGTAGC

Reverse, GGTGTGGGTGAGGAGCACAT

Gene Primer sequences (5’ to 3’)

*mM-csf* Forward, AACAGCTGCTTCACCAAGGA

Reverse, CCACATGGCCTCGGCTAGAG

*mMcp-1* Forward, CACTCACCTGCTGCTACTCA

Reverse, GCTTGGTGACAAAAACTACAGC

*mCcr2*  Forward, GCCATACCTGTAAATGCCATGC

Reverse, GCCGTGGATGAACTGAGGTA

*mNotch1* Forward, GCGAAGTGGACATTGACGAG

Reverse, GCTGGCACAGGCAGGTAAAG

*mNotch2* Forward, ACTGGGCAGCTGCTGTCAATAA

Reverse, AAGGCGGTCCATGTGGTCA

*mRbpj-κ* Forward, TTGGCGAGAGTTTGTGGAAGA

Reverse, AGCTTCCCTAGTGAGTCGTTTG

*mHes1* Forward, GCAGACATTCTGGAAATGACTGTGA

Reverse, GAGTGCGCACCTCGGTGTTA

*mHes5* Forward, AGTCCCAAGGAGAAAAACCGA

Reverse, GCTGTGTTTCAGGTAGCTGAC

*mSocs3* Forward, AGCTAATGAAACCTCGCAGATCC

Reverse, AGCTCACCAGCCTCATCTGTCTC
