# Supplementary figures and images for: Elevated Kallikrein-binding protein in diabetes impairs wound healing through inducing macrophage M1 polarization
Source: Cell Commun Signal. 2019 Jun 10;17:60. doi: 10.1186/s12964-019-0376-9 (PMC6558923; doi:10.1186/s12964-019-0376-9)

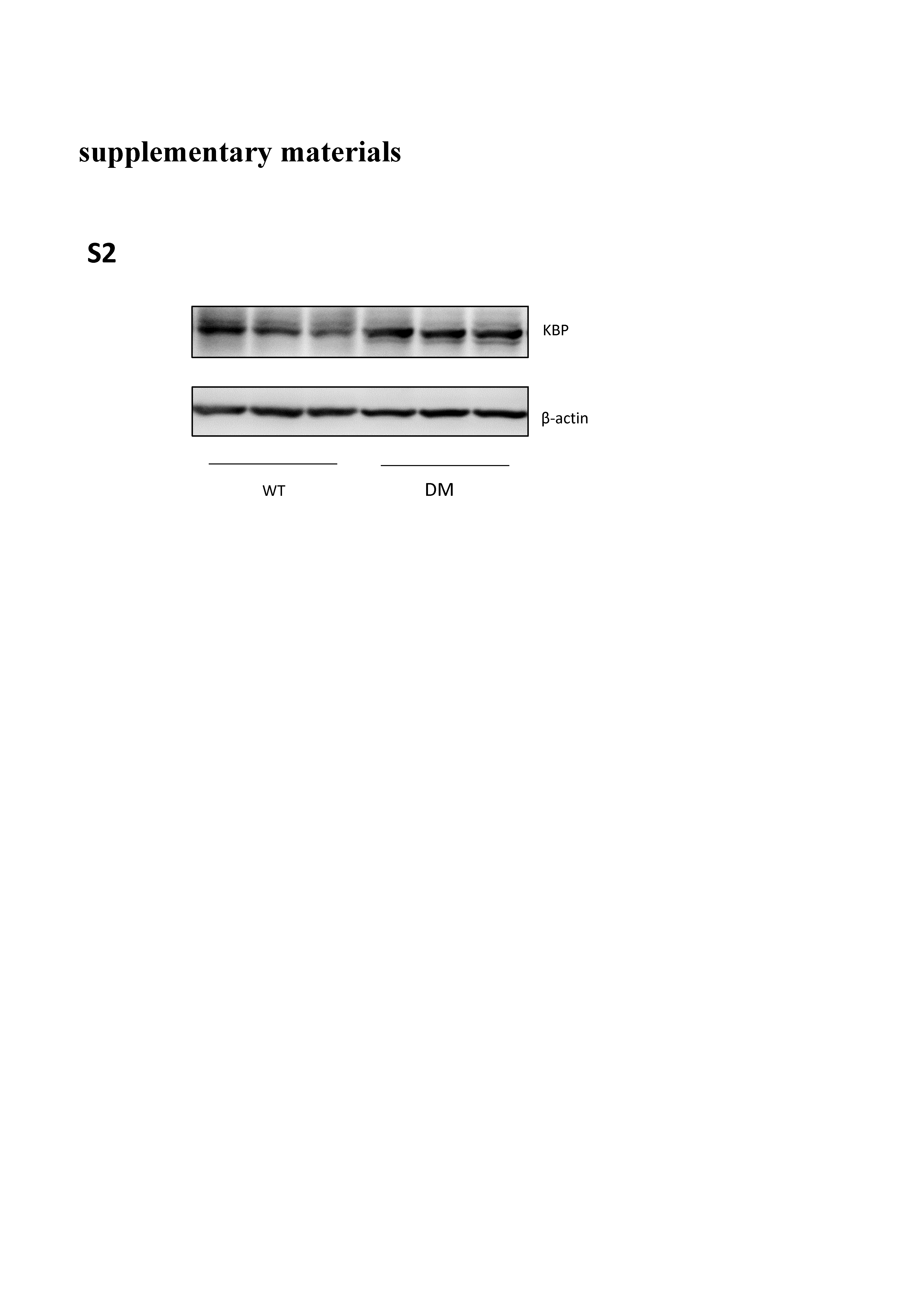

Supplement: Supplementary file 2 — Figure S2. The expression of KBP in the liver tissue of WT and diabetic mice (n = 3). (DOCX 214 kb) (TIFF 1695 kb) [file 12964_2019_376_MOESM2_ESM.tiff]

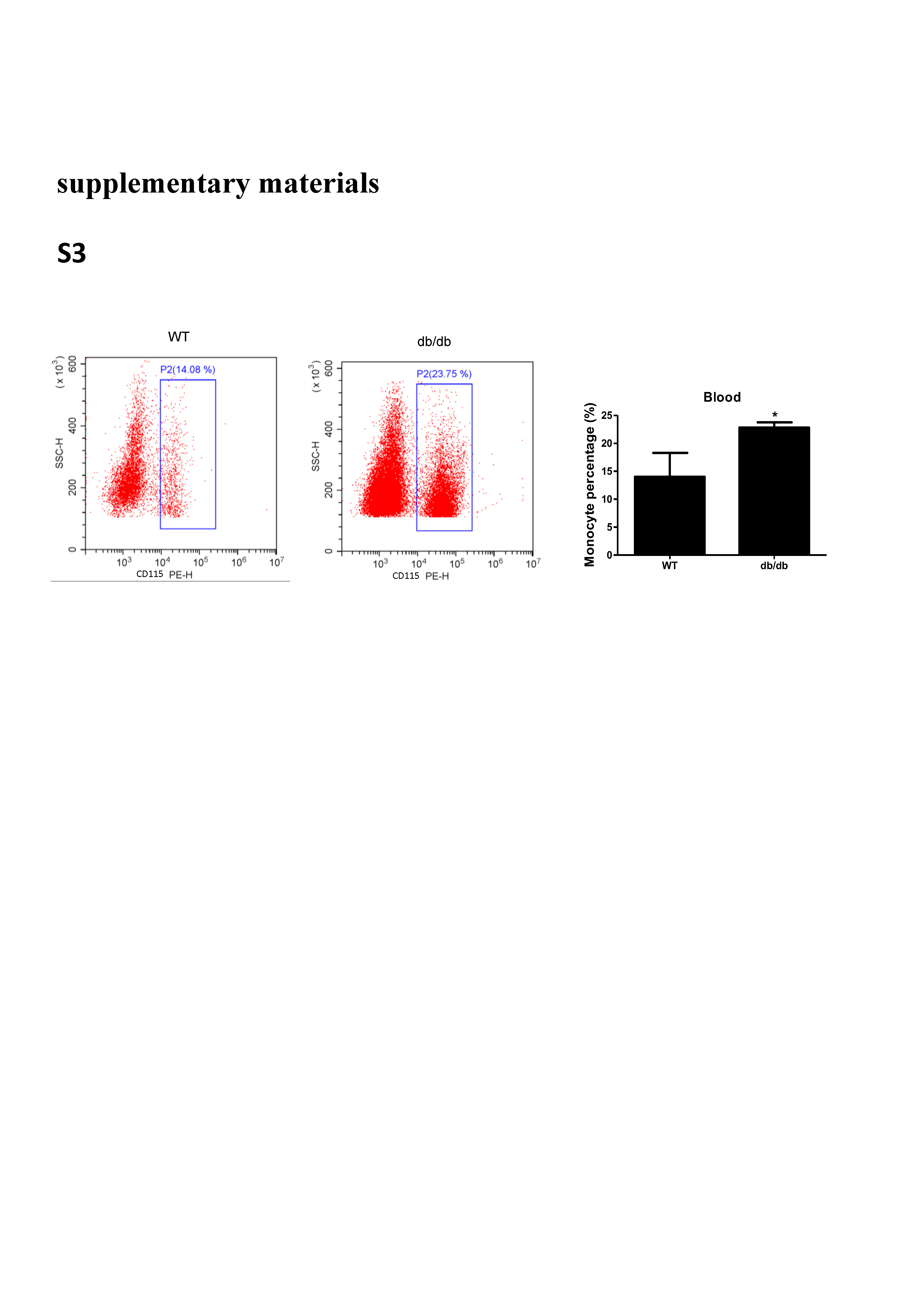

Supplement: Supplementary file 3 — Figure S3. Representative FACS results and quantification of CD115+ monocytes in peripheral blood of WT and db/db male mice. All data were presented as the mean ± SD. n = 3; * p < 0.05. (TIFF 2174 kb) [file 12964_2019_376_MOESM3_ESM.tiff]

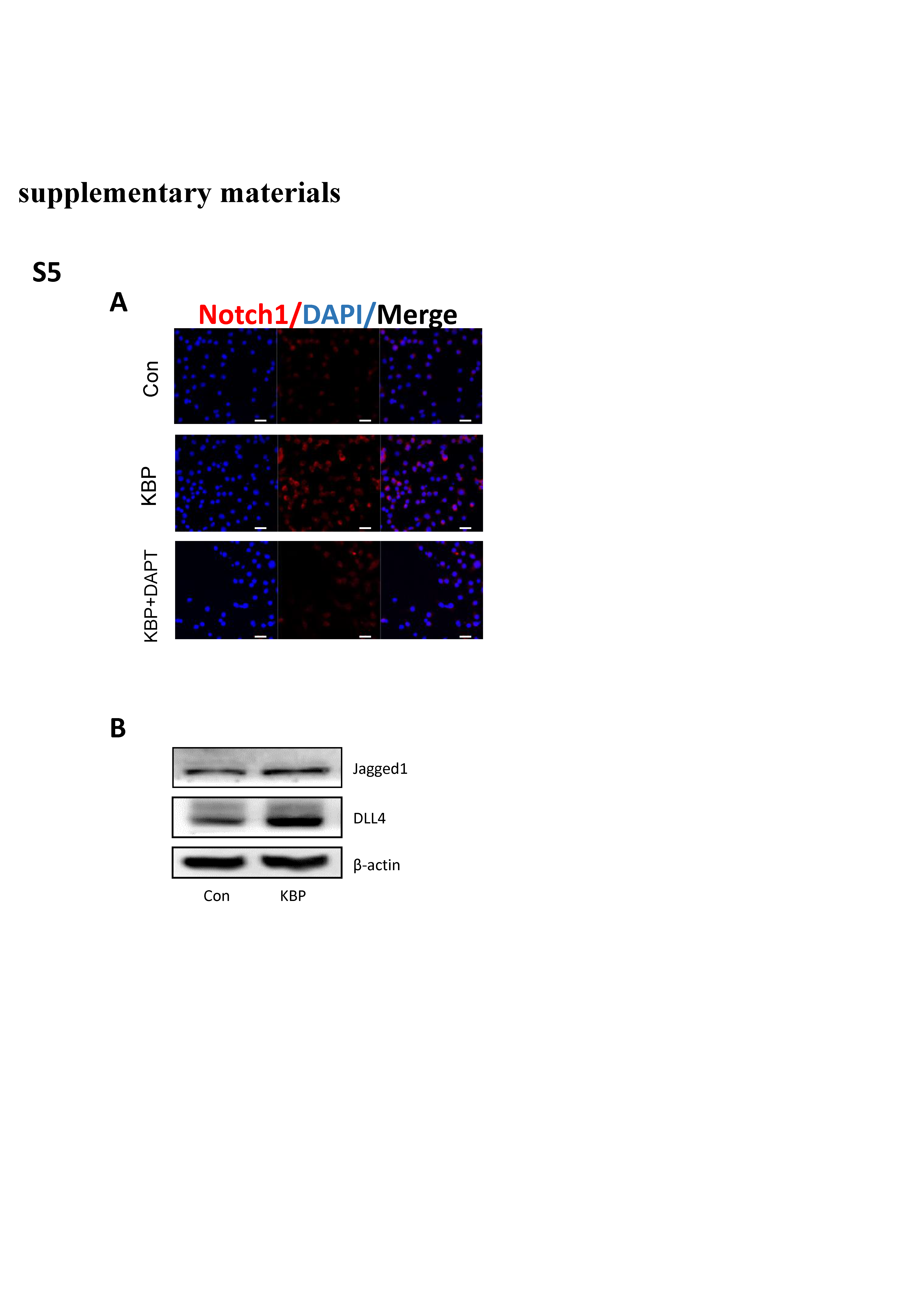

Supplement: Supplementary file 5 — Figure S5. A. The immunofluorescence staining results of notch1 in different groups of RAW264.7 macrophages. Scale bar = 5 μm. B. The expression of Jagged1 and DLL4 in RAW264.7 cells. (TIFF 4456 kb) [file 12964_2019_376_MOESM5_ESM.tiff]

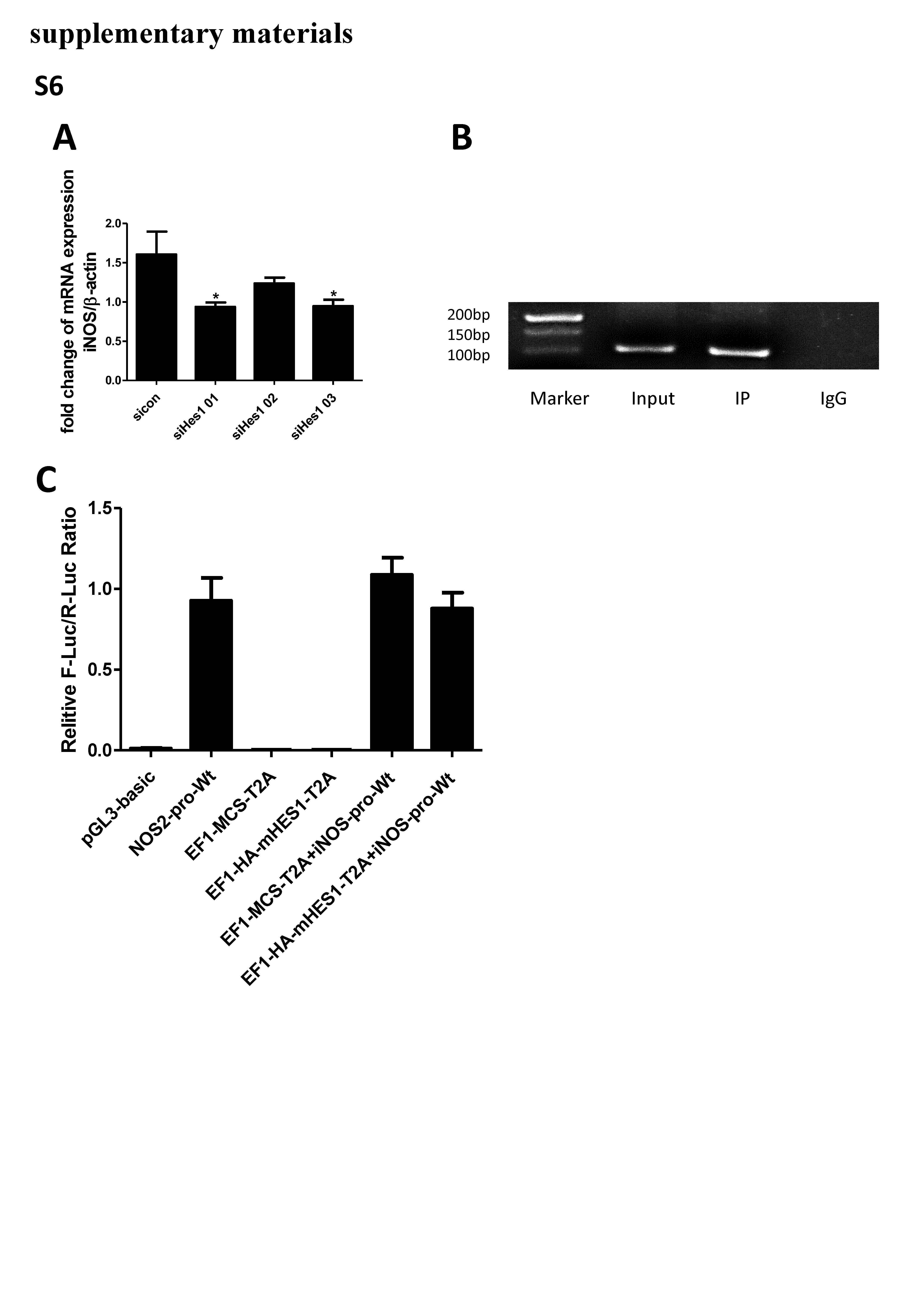

Supplement: Supplementary file 6 — Figure S6. A. The mRNA expression of iNOS in RAW264.7 cells after using sihes1. Data were presented as the mean ± SD. n = 3; * p < 0.05 versus sicon group. B. The ChIP assay of transcription factor hes1 and iNOS promoter region. C. The dual-luciferase report gene assay for hes1 and iNOS promoter in 293 T cells. (TIFF 1669 kb) [file 12964_2019_376_MOESM6_ESM.tiff]

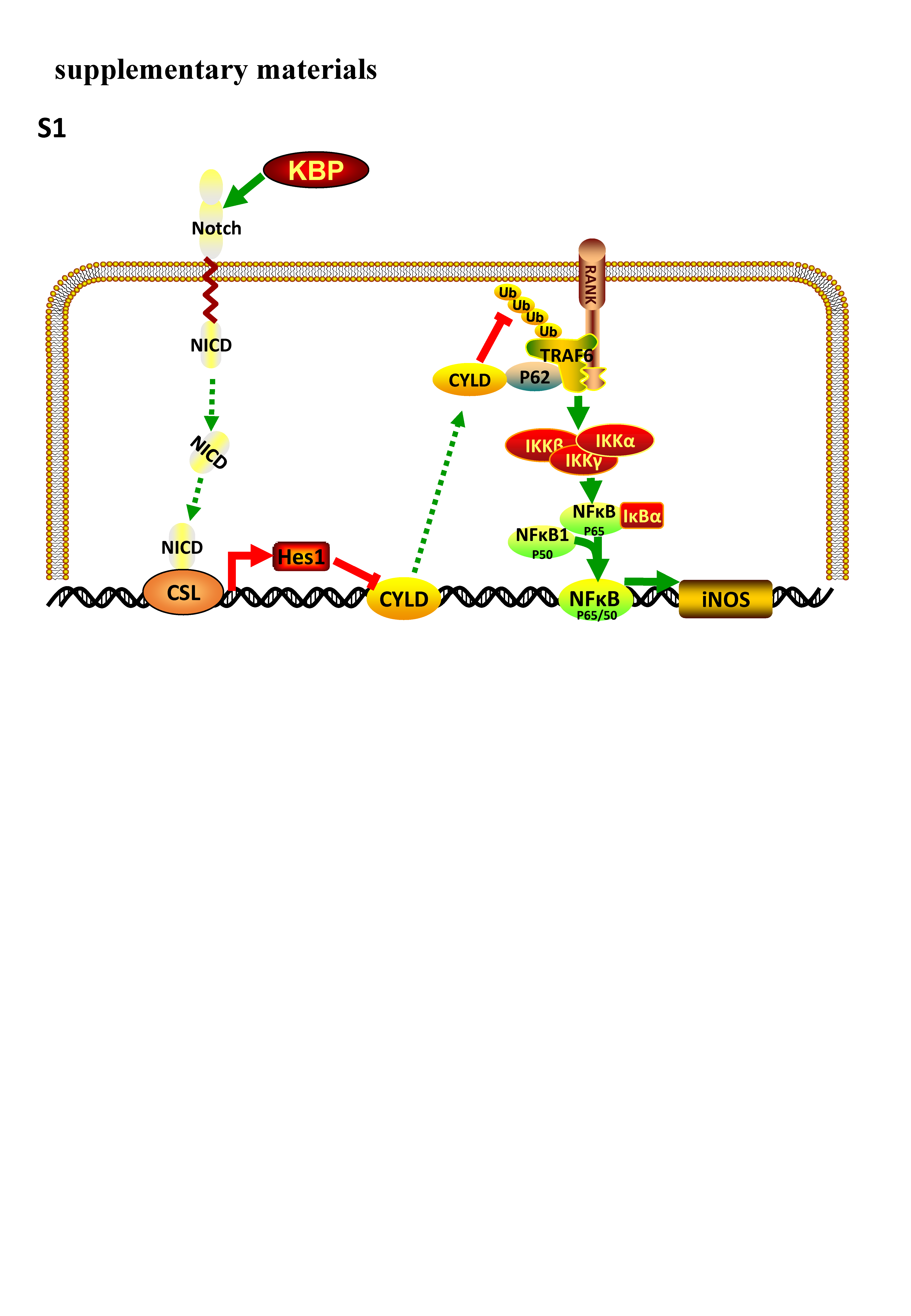

Supplement: Supplementary file 7 — Figure S1. The schematic overview of KBP in promoting the M1 polarization of macrophages by cross-activating the notch signaling pathway and NF-κB signaling pathway. KBP upregulates DLL4 and Notch1 to activate notch signaling, promoting NICD to bind to the CSL/RBPJ-κ, and then increases the expression of hes1. Hes1 suppresses the expression of CYLD, which could inhibit the ubiquitination of TRAF6. Because of the reduction of CYLD, the weakened deubiquitination of TRAF6 could activate NF-κB p65 via phosphorylation of IκBα. NF-κB p65 translocases into the nucleus to activate the expression of target gene-iNOS, to promote the M1 polarization of macrophages. (TIFF 2700 kb) [file 12964_2019_376_MOESM7_ESM.tiff]
